# The Mechanism of Boron–Carbon Bond Formation in the DA Reaction of the Pyridine Adduct of Borabenzene with Acetylene: A Topological Analysis of the ELF Function and Catastrophe Theory

**DOI:** 10.3390/molecules30112357

**Published:** 2025-05-28

**Authors:** Slawomir Berski

**Affiliations:** Faculty of Chemistry, University of Wroclaw, 14 F. Joliot-Curie, 50-383 Wroclaw, Poland; slawomir.berski@uwr.edu.pl; Tel.: +48-(0)71-3757246

**Keywords:** chemical bond, reaction mechanism, Diels–Alder, topology, electron localization function, ELF, BET

## Abstract

The mechanism of the DA cycloaddition reaction between the pyridine adduct of borabenzene and acetylene has been investigated using topological analysis of the electron localization function (ELF) and catastrophe theory (bonding evolution theory, BET). The study focuses on the differences in the electronic structures of C-C and C-B bonds during their formation. Additionally, the influence of electron density functionals with different constructions (B3LYP, CAM-B3LYP, B2PLYP, M06, M062X, and M052X) on the BET results was examined. The reaction proceeds through ten distinct phases. The B-C bond forms first, followed by the C-C bond. Significant differences were observed in the behavior of the non-bonding basins V(C) and V(B) compared to the V(C), V(C) basins, which precede the formation of the bonding basins V(B,C) and V(C,C). The use of different functionals results in quantitative variations in the lengths and positions of the reaction phases—for example, relative to the transition state structure. A possible qualitative influence on the overall picture of the reaction mechanism is suggested by the results obtained using the CAM-B3LYP and B2PLYP functionals, particularly in phases VI and VII.

## 1. Introduction

Diels–Alder (DA) cycloaddition reactions [1] are among the most frequently studied and described in the scientific literature [2,3,4]. From a mechanistic perspective, one of the most interesting problems is the question of their mechanism, particularly the order in which chemical bonds form. Are chemical bonds formed simultaneously during the reaction (concerted), or do they form successively in a specific order [5]?

Boron compounds have become the focus of many scientific studies in recent years [6,7] due to their use as pharmacophores and their promising applications in neutron capture therapies for cancer (boron neutron capture therapy) [8,9,10,11]. The effective use of boron compounds in DA and other synthetic reactions has been described in the works of Eberlin et al. [12], Pyziak et al. [13], and Welker [14].

While the mechanisms of DA reactions involving the formation of carbon–carbon (C-C) bonds have been extensively studied using computational quantum chemical methods [15], the formation of carbon–boron (B-C) bonds appears to be largely undescribed in the scientific literature.

**Scheme 1:**
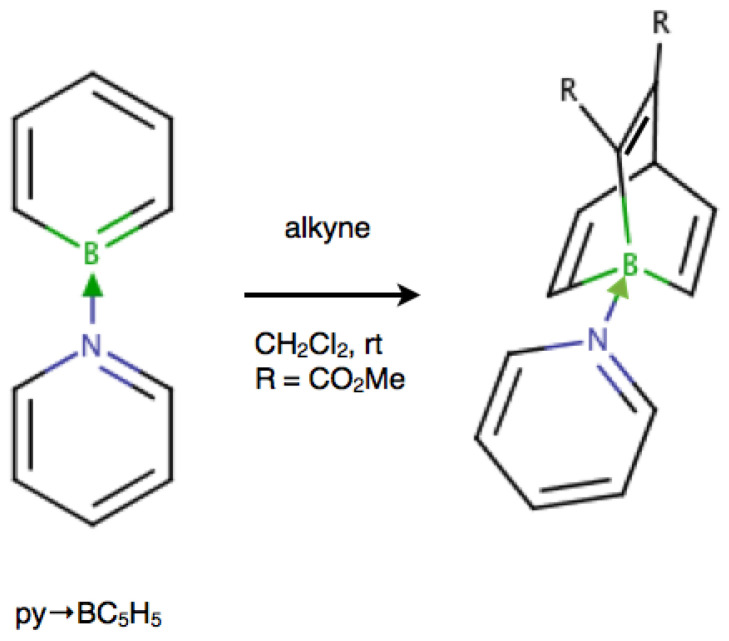
Lewis structures for the reaction of the pyridine adduct of borabenzene (py→BC_5_H_5_) with the bond-localized Kekulé representation of the aromatic system and the dienophile dimethyl acetylenedicarboxylate, as studied by Wood et al. [16].

**Scheme 2:**
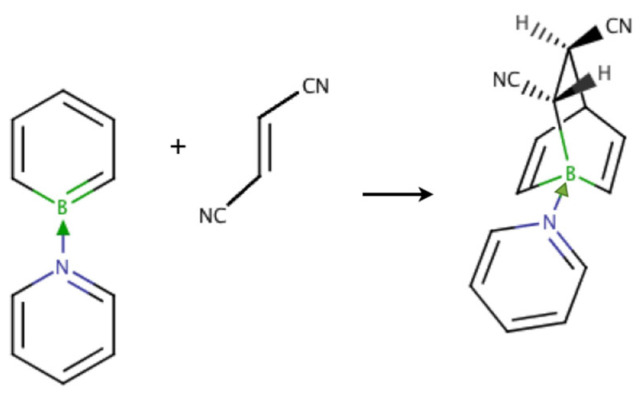
Lewis formulas for the reaction of py→BC_5_H_5_ with trans-1,2-dicyanoethylene studied by Wood et al. [16].

In 2006, Wood et al. [16] reported the results of an experimental study on the DA reaction of borabenzene (pyridine adduct, py→BC_5_H_5_) with the strong electrophilic dienophile dimethyl acetylenedicarboxylate, leading to the formation of a 1-borabarrelene derivative (see Scheme 1). In additional studies aimed at elucidating the DA reaction mechanism, the reaction product of py→BC_5_H_5_ with trans-1,2-dicyanoethylene was analyzed (see Scheme 2). The authors of this report claim they discovered a new type of reactivity in the class of heterocyclic compounds with a borabenzene skeleton, as demonstrated by the synthesis of 1-borabarrelene and 1-borabenzobarrelene derivatives [16].

To investigate the reaction mechanism from the perspective of chemical bond formation and breaking, a methodology is needed that precisely describes chemical bonding and identifies key points along the reaction path associated with bond formation and cleavage. In 1997, Krokidis et al. [17] introduced a new methodology for studying reaction mechanisms, based on the description of molecular electronic structure through topological analysis of the electron localization function (ELF) [18,19,20,21,22,23] with its evolution analyzed using elements of catastrophe theory (CT) [24]. This combination of computational quantum chemistry methods, gradient analysis of the ELF field, and catastrophe theory is known as bonding evolution theory (BET) [17].

The electron localization function (ELF) depends on the total electron density and provides a measure of the likelihood of finding an electron in the vicinity of a reference electron located at a given point and with the same spin [18]. ELF was defined by Becke and Edgecombe [18] based on the local behavior of the Hartree–Fock parallel spin electron pair probability. The catastrophe theory is a branch of geometry that studies how the equilibria of a gradient system evolve as the system’s control parameters change [17,24]. Readers interested in this topic should refer to the pioneering works of Prof. B. Silvi and his research group [25,26,27,28,29].

It is worth mentioning that the pioneer in the application of gradient field analysis in quantum chemistry was R. Bader [30], who showed that concepts such as atom and molecule should be related to the topological properties of the molecular electron density field [31]. In research using the quantum chemical topology [32] method, a change in electronic structure is associated with a change in the number and/or arrangement of bond paths, thus catastrophes in which continuous change in the nuclear coordinates result in a discontinuous change in a molecule’s behavior (topology of electron density field). Bader et al. [33] illustrated the application of CT to study the reaction mechanisms using the example of the formation of three-membered rings of H_3_^+^ and H_2_O molecules [34].

**Scheme 3:**
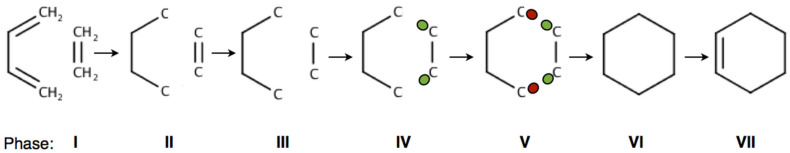
Modified Lewis formulas for the DA reaction of ethylene with *cis*-1,3-butadiene, adjusted according to the results of BET analysis. Each structure represents the topology of the ELF molecular field in a specific phase (structural stability domain) of the reaction. Lines denoting chemical bonds correspond to disynaptic attractors V(C,C). Green and red ellipses near carbon atoms represent monosynaptic, non-bonding V(C) basins. Diagram adapted from data in [35].

In 2003, we applied the BET methodology to investigate the prototypical DA cycloaddition reaction between ethylene and 1,3-butadiene [35] and later reactions between 1,3-butadiene and acrolein, and 2,4-pentadienal and methyl vinyl ether [36]. Among other findings, we demonstrated that the formation of the reaction product is associated with a series of ELF field catastrophes, consisting of seven phases corresponding to distinct structural stability domains, SSD (see Scheme 3). We also showed that, at the transition state structure (TS), no C-C chemical bonds are being formed or broken, and that the overall reaction mechanism is not concerted in nature.

The first observed process (phase II) is the “reduction” in the double C=C bonds to single bonds in the butadiene molecule, followed by the “reduction” in the C=C bond in ethylene (phase III). Next, electron density becomes concentrated in the regions where the new C-C bonds will form (phases IV and V). The intermolecular C-C bonds are formed in phase VI, which occurs after the transition state structure has been passed. At the end of the reaction (phase VII), a new double C=C bond is formed—i.e., the local topology of the ELF field becomes similar to that observed for the carbon–carbon bond in the H_2_C=CH_2_ molecule. The initially identified fold (F) and cusp-type catastrophes were later revised and simplified to fold-type only in a more detailed and thorough study [37].

Building on the existing knowledge of C-C bond formation mechanisms in DA cycloaddition reactions [35,36], it is particularly interesting to investigate the mechanism of electron density redistribution—specifically, the breaking and formation of chemical bonds—when one of the interacting atoms is boron (i.e., comparing C-C vs. B-C bond formation).

**Scheme 4:**
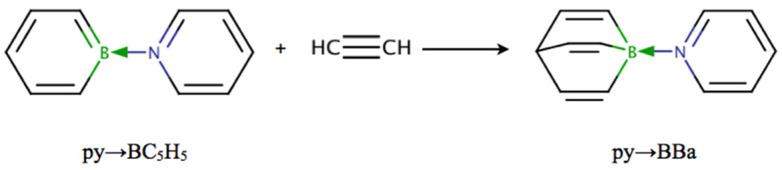
Lewis formulas illustrating the reaction of the pyridine adduct of borabenzene (py→BC_5_H_5_) with acetylene (HC≡CH), yielding the pyridine adduct of 1-borabarrelene (py→BBa). For the BC_5_ and NC_5_ rings, bond-localized Kekulé representations are used to depict the delocalized π-systems.

This study focuses on a simplified system inspired by the results of Wood et al. [16], specifically the reaction of py→BC_5_H_5_ with acetylene to form the pyridine adduct of 1-borabarrelene (py→BBa): py→BC_5_H_5_ + HC≡CH→py→BBa (see Scheme 4).

An additional motivation for investigating the reaction of py→BC_5_H_5_ with HC≡CH stems from the fact that, while we have relatively extensive information about the electronic structure of individual boron compounds from an ELF topological perspective [38], we know relatively little about the behavior of their boron bonds during processes involving changes in bond length. In a series of studies on B-B [39], B-C [40], B-N [41,42], B-O, B-F (B-Cl), and B-Cu bonds (See reference [39] and other articles cited therein), we have shown that in the case of B-C and B-N bonds, it is possible to observe single, double, and triple bonds, in line with classical representations of these bonds (2, 4, and 6 electrons, respectively), such as in Lewis formulas that account for an octet of electrons.

**Figure 1:**
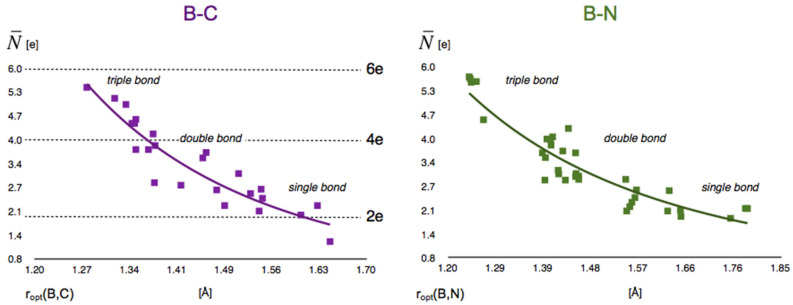
The relationship between the populations of the V(B,C) and V(B,N) basins and bond lengths, r_opt_(B,C), and r_opt_(B,N), for molecules with BC and BN bonds investigated in references [40,42]. The graphs were prepared based on data from these studies, with computations performed at the DFT(M062x)/aug-cc-pVTZ, DFT(M062x)/Def2-TZVPD (BC) and DFT(M062X)/6-311+G(d,p) (BN) levels.

Furthermore, we have shown that the population of the V(B,N) basin, calculated for 25 organo-boron molecules, ranges from 5.72e to 1.83e, while for the V(B,C) basin, calculated for 19 molecules, the range is between 5.46e and 1.22e (see Figure 1). Analysis of the contributions to the population of the V(B,N) basin from quantum atoms in molecules of varying lengths has shown that the primary change is due to the contribution of the N atom [42]. It is of interest to verify this observation for the process of B-C bond formation and breaking, as previous studies of single molecules [40] also indicate that the electron density from the C atom dominates its nature.

To summarize, the calculations presented in this paper were undertaken to answer the following questions:

What is the mechanism of the cycloaddition between py→BC_5_H_5_ and HC≡CH, using the concepts of bond formation and breaking?

Is there a qualitative difference in this mechanism compared to the prototypical DA reaction between H_2_C=CH_2_ and CH_2_=(CH)_2_=CH_2_?

Is the B-C bond formed through the sharing of electron density between the B and C atoms, or is it a donor mechanism, with valence electron density transferred from the C atom to the B-C bonding region? With the full involvement of the 2p orbitals from the B atom in the formation of B-C bonds and the stabilization of the molecule by the lone pair of pyridines, which forms a B→N bond using the unoccupied 2p orbital, the question arises: does the necessary rehybridization of orbitals on the B atom enable the formation of a covalent-polarized bond or a donor–acceptor bond (B←C)?

Is there any significant difference in the evolution of chemical bonds in the investigated reaction when studied using wave functions approximated by the DFT method with electron density functionals from the following different groups: B3LYP, CAM-B3LYP, M052X, M06, M062X, and B2PLYP?

Due to the large number of available electron density functionals [43], any arbitrary choice can be questioned; therefore, the study was limited to only six functionals. B3LYP [44] is a classical functional that achieved significant success, while M062X [45] from Truhlar’s group is considered the successor to B3LYP. The Minnesota functionals M06 [45] and the older M052X [46] were also considered. M06 and M062X are recommended for main-group thermochemistry and non-covalent interactions, and as global hybrid functionals, they contain 27% and 54% HF exchange, respectively. Our reacting system includes a B atom from the main groups, and the reaction course is undoubtedly associated with non-covalent interactions. The B2PLYP functional [47] is based on a mix of exchange and correlation with Hartree–Fock and a perturbative second-order correlation term (PT2), using DFT virtual orbitals [48]. The CAM-B3LYP functional, proposed by Yanai et al. [49], is a hybrid exchange–correlation functional that employs the Coulomb-attenuating method, which includes a correction for long-range interactions.

The article is structured as follows: Section 2.1 discusses the energetics of the studied reaction in the context of the applied DFT functionals. Section 2.2 describes the methodology of the topological analysis of ELF functions, as developed in the laboratory of Prof. B. Silvi. In Section 2.3, the electronic structure of the reactants and product is presented from the ELF topological perspective. Section 2.4 provides a detailed discussion of the reaction mechanism, divided into individual phases. Section 3 and Section 5 present the discussion of the obtained results and the final conclusions drawn from the conducted research.

In summary, inspired by the results of Wood et al. [16] and using our experience regarding the nature of C-C, B-C, and B-N bonds from an ELF topological perspective, I investigated the reaction mechanism of py→BC_5_H_5_ with HC≡CH in order to explain the differences in the formation of B-C and C-C bonds.

## 2. Results

### 2.1. The Stationary Points and Energetic Parameters

**Figure 2:**
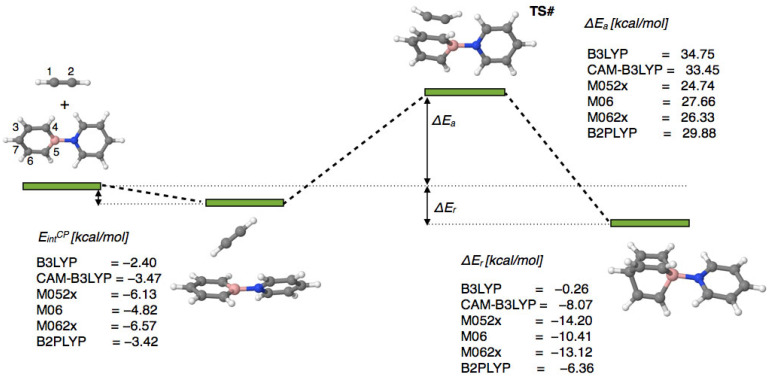
The energetic parameters (interaction energy, E_int_^CP^, activation energy, ΔE_a_, and reaction energy, ΔE_r_) and optimized geometrical structures for the stationary points in the reaction between py→BC_5_H_5_ and HC≡CH. The interaction energy has been corrected for the basin set superposition error (CP). The ΔE_a_ and ΔE_r_ energies include corrections for the difference in zero-point vibrational energies (ΔZPVE).

The DFT-optimized structures of the reactants, transition state structure, and product of the reaction are presented in Figure 2. The reaction between the py→BC_5_H_5_ and HC≡CH molecules, studied in the gas phase (vacuum), begins with the formation of a molecular complex (MC) py→BC_5_H_5_⋯HC≡CH, stabilized by weak hydrogen bonds. The interaction energy of the MC (E_int_^CP^) varies depending on the electron density functional used, ranging from −2.40 kcal/mol (B3LYP) to −6.57 kcal/mol (M062X).

The activation energy, ΔE_a_, calculated with respect to the sum of isolated reactants, ranges from 24.74 kcal/mol (M052X) to 34.75 kcal/mol (B3LYP). The highest activation barrier values (>33 kcal/mol) are obtained using the B3LYP and CAM-B3LYP functionals (33.45 kcal/mol). The inclusion of the long-range interaction correction (CAM) in the B3LYP functional reduces its activation energy by 1.3 kcal/mol. The three functionals based on the meta-GGA approximation from Truhlar’s group (M052X, M06, and M062X) yield ΔE_a_ values within a relatively narrow range of 24.74–27.66 kcal/mol, which are smaller than those obtained using the B3LYP functional. The B2PLYP value (29.88 kcal/mol) falls between these two groups. The relatively high activation barrier calculated, for example, by the B3LYP method (ca. 35 kcal/mol) suggests that its occurrence under experimental conditions is very unlikely. This is due to the marginal electrophilic character of the HC≡CH molecule.

The reaction product lies below the reactants on the total energy axis (ΔE_r_, −0.26 kcal/mol to −14.20 kcal/mol), indicating the possibility of an exothermic process. The relatively large range of values suggests a significant dependence of ΔE_r_ on the choice of functional. Notably, the low value calculated using the B3LYP functional (−0.26 kcal/mol) is significantly affected by the CAM correction, which reduces the value to −8.07 kcal/mol (CAM-B3LYP). Similar to the activation barrier values, the functionals from Truhlar’s group give results within a relatively narrow range: −10.41 kcal/mol (M06) to −14.20 kcal/mol (M052X).

In the transition state structure, the HC≡CH molecule is located near the py→BC_5_H_5_ molecule at a distance, measured between the C_1_,C_7_ and C_2_,B atoms, that is smaller than the sum of the van der Waals radii of the interacting atoms. The optimized values of r(C1,C7) and r(C2,B), calculated using different electron density functionals, are presented in Table 1. Comparison of these values shows that introducing a correction for long-range interactions (CAM) to the B3LYP functional (CAM-B3LYP) alters the description of the C1⋯C7 and C2⋯B interactions. The r(C1,C7) distance in the TS is slightly shortened by 0.020 Å, while the r(C2,B) distance is lengthened by 0.033 Å compared to the B3LYP value. Similarly, the three functionals based on the meta-GGA approximation from Truhlar’s group (M052X, M06, and M062X) describe the C1⋯C7 and C2⋯B interactions differently. Changing from M052X to M062X results in a lengthening (0.019 Å) of r(C1,C7) and a shortening (0.046 Å) of r(C2,B). In contrast, the use of the hybrid functional M06, designed for non-covalent interactions and organometallic compounds, produces the largest r(C1,C7) value and the shortest r(C2,B) distance among all the functionals used, measuring 2.352 Å and 2.076 Å, respectively. Finally, applying the double-hybrid functional B2PLYP, which incorporates the PT2 correlation for exchange-correlation energy calculation, yields a significantly larger r(C1,C7) distance of 2.307 Å compared to most of the other functionals (B3LYP, CAM-B3LYP, M052X, and M062X). A similar trend is observed for the r(C2,B) distance, with a value of 2.132 Å, which is notably smaller than those calculated with the other functionals (see Table 1).

As mentioned, it will be interesting to assess how the selected functionals, particularly M06 and B2PLYP, influence the description of the reaction mechanism using BET.

### 2.2. Topological Analysis of ELF Function in Molecules—Outline of the Methodology

The reaction under study involves the py-BC_5_H_5_ and HC≡CH molecules. As an introduction to the methodology of topological analysis of the ELF function in molecules, the electronic structure of HC≡CH will be discussed first. Interested readers are referred to the works of Prof. Silvi’s group. Unless otherwise stated, all results presented are based on DFT(M062X)/6-311++G(d,p) calculations.

The acetylene molecule consists of 14 electrons that move (in the classical representation of the problem) in an electrostatic potential field generated by 14 positively charged atomic nuclei and negatively charged electrons. The total electron density can be characterized as belonging to the non-reacting regions of the atomic cores (in this case, the carbon atoms) and to the valence space, which undergoes significant redistribution during chemical processes. 

The ELF function, being a measure of electron localization at a given point in space, changes its values from 0 to 1 [18]. The ELF gradient field analysis allows for the identification of four types of critical points, the most important of which are local maxima (attractors). As shown by Silvi and Savin [19,20], these correspond to core electrons, lone pairs of electrons, and chemical bonds [20]. Thus, the elements of the electronic structure of molecules predicted in Lewis representations [50] have been rigorously confirmed by the ELF topological analysis [19].

**Figure 3:**
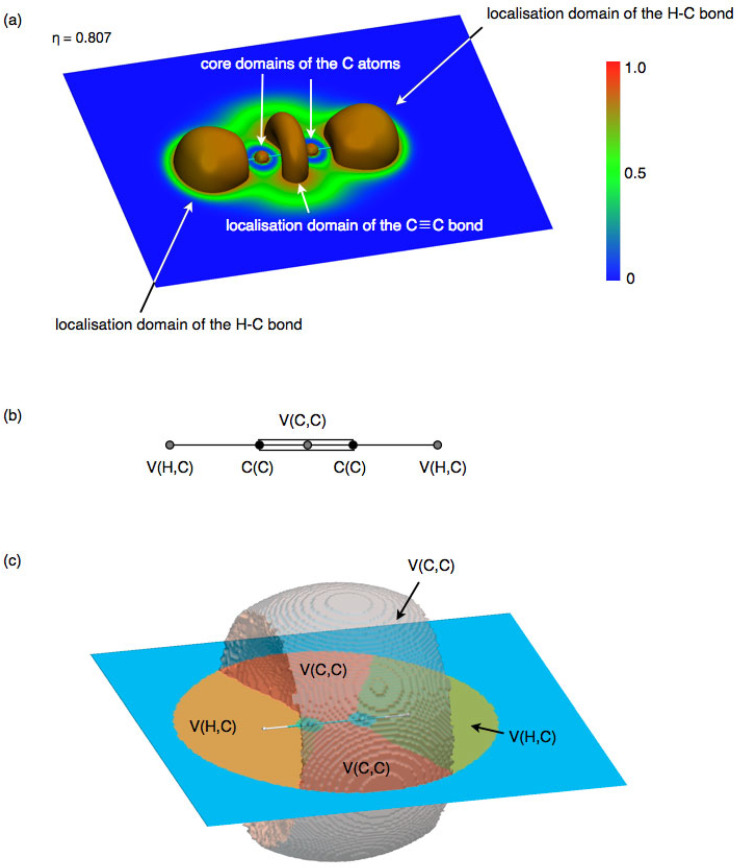
(**a**) A three-dimensional representation of the distribution of ELF values (η) in an HC≡CH molecule, superimposed on a two-dimensional cross-section in the plane containing all atoms. The ELF function varies between 0 and 1, where 1 represents perfect localization of electrons. (**b**) Positions of the core and valence attractors of the ELF molecular field in HC≡CH, superimposed on the Lewis diagram. (**c**) A three-dimensional representation of the localization basins associated with the ELF field attractors, superimposed on a two-dimensional plane slice containing all the atoms. The attractor and basin designations are as follows: C—core, V—valence. The analysis was performed using DFT(M062X) data.

The first step involves a visual inspection of the distribution of ELF values across topological manifolds, called localization domains (ELF-localization domains), which are visible for a given ELF (cutoff) value. The localization domains corresponding to the carbon cores, the H-C bonds, and the C≡C triple bond are shown in Figure 3a. The domains corresponding to the atomic core regions are clearly visible for high ELF values, which characterize highly localized electron density. The valence localization domain observed between the core domains indicates the covalent carbon-carbon bond. The high degree of electron localization visible outside the bond axis is usually interpreted as a manifestation of the multiple nature of the C≡C bond, which is related to the overlap of orbitals in a π fashion.

In the second step of the analysis, the electronic structure description is typically refined through gradient analysis of the ELF field and the locations of attractors. In the HC≡CH molecule, the core regions of the carbon atoms are characterized by two attractors (ELF local maxima) designated as C(C) and C(C). In the valence space, two H-C bonds and a formally triple C≡C bond are described by three valence attractors: V(H,C), V(H,C), and V(C,C). Since these attractors are associated with valence electron density identified with chemical bonding regions, they are classified as bonding and disynaptic [21]. All attractors in HC≡CH take the geometrical form of points. The locations of these attractors in molecular space are shown in Figure 3b. It is noteworthy that for the carbon-carbon triple bond, the topological analysis reveals only one local maximum at the midpoint of the C≡C bond. At this stage of the analysis, there is some agreement with the classical Lewis representation of the HC≡CH molecule’s electronic structure. The core and valence attractors are positioned where the atomic cores and chemical bonds (H-C and C≡C) are expected. According to Silvi and Savin [19], the localization of bonding attractors serves as evidence that a chemical bond has covalent character.

Each ELF field attractor is associated with a localization basin, which precisely defines the points in space where the ELF value is calculated, “contributing” to the attractor’s basin (inset). The names of the localization basins correspond to the names of the attractors, and they are classified into types: bonding, nonbonding, core, and valence. In the HC≡CH molecule, core basins C(C), C(C), and valence basins V(H,C), V(H,C), and V(C,C) are observed. Figure 3c shows a cross-section through the localization basins in the plane of the molecule, along with the three-dimensional representation of the basin associated with the attractor V(C,C). The analysis of the distribution of the V(C,C) basin in space provides insight into the formal structure of the C≡C triple bond.

The nature of chemical bonds is fully described by the analysis of the electron population in localization basins ($\bar{N}$). Consistency with the classical model based on Lewis theory, as well as with results from analyses using the orbital approximation of the wave function, is expected. The $\bar{N}$ values for the basins in HC≡CH are summarized in Table 2. To investigate the influence of the chosen electron density functional, the basin populations obtained using the following methods were compared: B3LYP, CAM-B3LYP, M052X, M06, M062X, and B2PLYP. The comparison shows that the influence of the density functional, in this case, is negligible.

Core basins with a population of approximately 2.1e are in excellent agreement with the theoretical value derived from the electronic configuration of the K-shell core electrons (1s^2^). The C≡C triple bond, represented by the V(C,C) basin, has a basin population ranging from 5.11 to 5.17e, which differs from the formal value of 6e. However, strict quantitative compliance with formal values (1e, 2e, 4e, and 6e) should not be expected, as this was already addressed in the early development of this methodology [20]. It appears that for multiple bonds, the discrepancy from formal values increases with the multiplicity of the bond. The “missing” electron density, in comparison to the formal value of 6e, can be found in the valence space of the H-C bonds. The population of the V(H,C) basins, which ranges from 2.32e to 2.35e, is clearly greater than the 2e expected for a formal single bond, further confirming the presence of a single H-C bond in the acetylene molecule. A larger-than-expected $\bar{N}$ value may be associated with the acidic properties of acetylene through the resonance of Lewis structures: H^−+^C=CH, HC=C^+^H^−^, and HC≡CH. The negative charge on the hydrogen atom facilitates the easier removal of a proton. This equilibrium of resonance structures also explains the population of the carbon-carbon bond being smaller than 6e.

The final element of the electronic structure description is the analysis of the atomic (quantum atom) contributions to the bonding basins. This approach to understanding the “structure” of localization basins was proposed by Raub and Jansen [51]. Each V(H,C) basin consists of 0.81e contributed by the hydrogen atom (35%) and 1.51e contributed by the carbon atom (65%). As shown in ref [51], these values can be used to calculate the bond polarity index, p_AB_, for the A-B bond, which takes values in the range of 0 (homopolar bonds) to 1 (idealized ionic bonds). The higher the index value, the greater the bond polarity, which results from a larger contribution of electron density from one of the A or B atoms to its formation. The covalent-polarized H-C bonds are dominated by the electron density from the C atom, and the p_CH_ index is 0.30. In the case of the covalent C≡C bond, the contributions of both carbon atoms are the same, so the index value is 0, indicating that the bond is not polarized.

A complementary aspect of the discussion regarding the electronic structure of molecules, which is especially important when describing the delocalization of electron density, is the analysis of the covariance (cov) values of basin populations [22,23]. In the HC≡CH molecule, the C≡C bond density is significantly delocalized with the two H-C bonds, as indicated by the covariance values cov[V(C,C),V(H,H)] = −0.53, and to a lesser extent with the core C(C) basins, as shown by the covariance values cov[V(C,C),C(C)] = −0.16.

The above-described method of analyzing the results obtained from the topological analysis of the molecular ELF field will be applied to all molecular structures discussed in this work.

### 2.3. Electronic Structure of Reactants and Product Molecules

**Figure 4:**
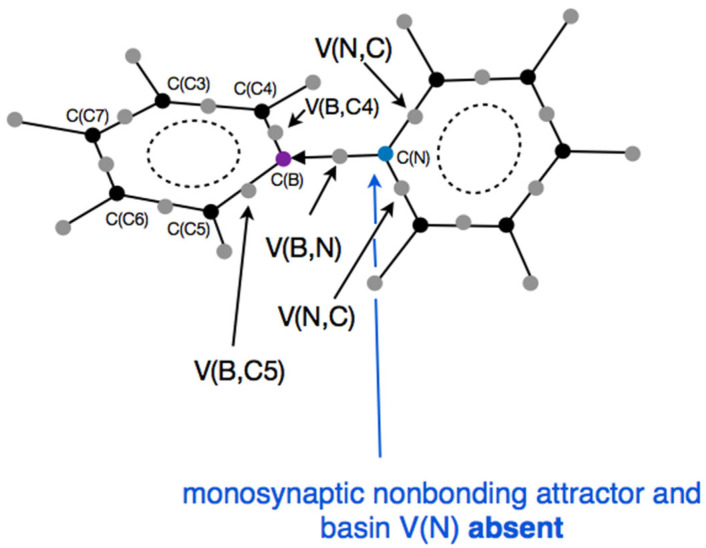
The core and valence attractors in the py→BC_5_H_5_ molecule. The analysis using DFT(M062X) data.

The py→BC_5_H_5_ molecule (the pyridine adduct of borabenzene) is a perfect example of a system with a covalent dative boron-nitrogen (B←N) bond. Its electronic structure, represented by the local maxima of the molecular ELF field, consists of 35 core and valence attractors. All attractors, shown in Figure 4, are of the point type. There are no nonbonding attractors in the molecule, which would indicate the presence of lone electron pairs, particularly in the valence space of the nitrogen atom.

The 12 non-bonding core attractors characterize the electron density in the atomic cores of the carbon (C(C)), boron (C(B)), and nitrogen (C(N)) atoms. The 23 bonding attractors in the valence space represent the chemical bonds: 10 protonated disynaptic attractors, V(H,C), correspond to the H-C bonds; 12 disynaptic attractors correspond to 1 B←N bond (V(B,N)), 2 B^…^C bonds (V(B,C4), V(B,C5)), 2 N-C bonds (V(N,C)), and 8 C^…^C bonds (V(C,C)). These bonding attractors provide evidence that the chemical bonds they represent have a covalent character, with electron density shared by both atoms.

In the two XC_5_ (X = B, N) rings, only bonding attractors—V(C,C), V(B,C), and V(N,C)—are observed in the plane of the rings. This localization of attractors aligns with the expected effective delocalization of the ring’s electron density. In molecular systems where aromatic properties are not in question, the attractors representing chemical bonds in the ring are typically located precisely within the plane (e.g., benzene [20]). On the other hand, well-localized C=C double bonds in rings, which may suggest the absence of aromatic properties, are represented by valence attractors, V_i_(C,C), that are localized above and below the plane of the ring, as seen in the cyclohexene molecule (see Supplementary Material, Figure S1).

The dative B←N bond is represented by the V(N) monosynaptic attractor and basin, which is localized along an imaginary line joining the C(B) and C(N) attractors. Formally, its non-bonding character does not provide direct evidence that the boron-nitrogen bond has covalent character, with electron density shared by both atoms. Instead, it clearly demonstrates that the bonding arises from the donation of the lone electron pair from the N atom (V(N)) to the valence space of the B atom. This ELF representation aligns well with the classical understanding of the nature of a covalent dative bond. However, one should note that the identification of synapticity in donor–acceptor bonds may be subject to some imprecision due to numerical procedures. It is likely that the same bond could also be described by a disynaptic bonding basin, V(B,N), with electron density originating solely from the N atom, which, in both cases, leads to the same overall interpretation.

**Figure 5:**
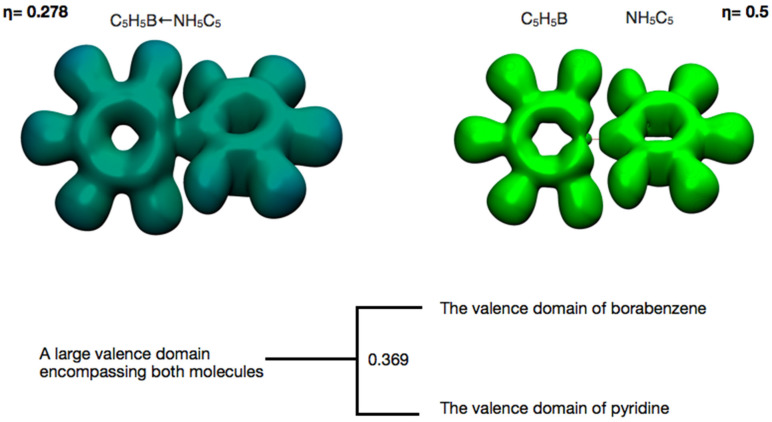
Representation of the distribution of ELF function values in the py→BC_5_H_5_ molecule for η = 0.278 and η = 0.5 (electron gas). For η = 0.278, a single reducible localization domain encompassing the whole molecule is visible, while for η = 0.5, two clearly separated reducible localization domains are distinguishable, each corresponding to the borabenzene (BC_5_H_5_) and pyridine (py) molecules, which interact via the B←N bond. The analysis was performed using DFT(M062X) data.

The donor–acceptor character of the interaction is also well reflected in the analysis of the reduction in localization domains as the ELF value changes from 0 to 1. Figure 5 shows the valence domains for ELF values of 0.278 and 0.5. At lower ELF values, a large superdomain is visible, encompassing both the pyridine and borabenzene molecules. As the ELF value increases to 0.369, this superdomain splits into two separate molecular domains, one for each interacting molecule. In the region of the B←N bond, the distribution of ELF values differs near the C(B) core compared to the C(N) core. Near the C(N) atom, a valence localization domain is visible, which represents the lone electron pair of nitrogen, while the ELF values near C(B) are notably smaller, forming a cavity in the local shape of the valence domain. For an ELF value of 0.5 (see Figure 5), a fragment of the C(B) domain can still be observed. In summary, the relatively low ELF value at which the cleavage of the superdomain occurs for the entire molecular adduct reflects the local electronic structure typical of a donor–acceptor covalent bond.

The basin population of the B←N bond is 2.55e, suggesting that it can be considered a single bond; although, from the perspective of valence bond theory, a contribution from a resonance form with a double bond should probably be taken into account. The V(N) basin consists of 2.43e from the nitrogen atom and 0.11e from the boron atom. Therefore, the B-N bond is 96% determined by the electron density originating from the nitrogen atom, classifying it as a donor–acceptor bond.

The boron–carbon bonds, represented by the V(B,C4) and V(B,C5) basins, have populations of 2.90e. These values, close to 3e, suggest an intermediate character between single and double bonds, indicating significant electron density delocalization within the BC_5_ ring. The atomic composition of these bonds is 2.37e (from carbon) and 0.50e (from boron), and the polarity index, p_CB_, is 0.65. Thus, the covalent-polarized B-C bonds are formed predominantly by electron density from the carbon atom (82%), indicating a relatively high degree of polarization. The next four C^…^C bonds in the BC_5_H_5_ ring have pairwise basin population values of 2.70e, 2.70e, and 2.78e, 2.78e. These values further suggest a delocalized nature for the C^…^C bonds, which cannot be definitively classified as single bonds. In the NC_5_ ring, the C-N bonds, with populations of 2.44e, deviate less from the formal value of 2e (by 0.44e) compared to the B-C bonds (0.90e), and can thus be described as single bonds.

The electron density of the V(N,C) basins consists of 1.90e from the nitrogen atom (78%) and 0.54e from the carbon atom (22%). The polarity index, p_NC_, is 0.56. Notably, the polarity index of the N-C bond is significantly smaller compared to the B-C bond. The next four carbon-carbon bonds in the NC_5_ ring have pairwise populations of 2.93e, 2.93e, and 2.70e, 2.70e. These values, close to 3e, indicate delocalization of electron density within the ring, suggesting that the C^…^C bonds are intermediate between single and double bonds.

The B-C and N-C bonds exhibit certain similarities. In both cases, the B and N atoms are, respectively, less and more electronegative than the C atom (Allred–Rochow, χ_B_= 2.01, χ_N_ = 3.07 vs. χ_C_ = 2.50). This difference is reflected in the contributions of the quantum atoms to the bonding basins. In both bonds, the contribution of the more electronegative atom is greater: the N atom (78%) in the V(N,C) basins and the C atom (82%) in the V(B,C4) and V(B,C5) basins.

**Figure 6:**
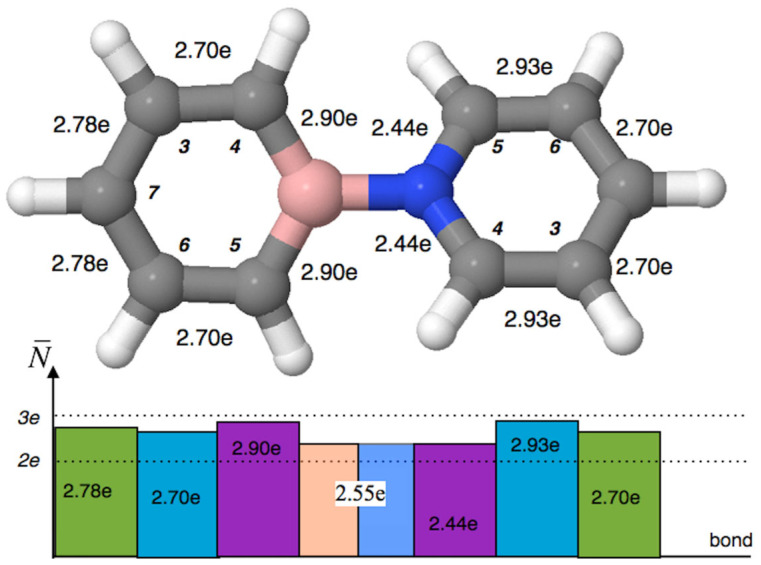
Comparison of electronic population values ($\bar{N}$ in e) for the bonding basins corresponding to the bonds in the BC_5_ and NC_5_ rings, as well as the B←N bond in the py→BC5H5 molecule. The analysis was performed using DFT(M062X) data.

The comparison of the population of carbon-carbon bonds in the NC_5_ ring relative to the BC_5_ ring yields interesting results (see Figure 6). Due to the differences in the physical properties of the B and N atoms, such as electronegativity, different polarization of the electron density in the ring is expected. Indeed, this difference is observed: in the borabenzene ring, the bonds to the B atom (B-C4, B-C5) are most saturated with electron density (2.90e), whereas in the pyridine ring, the bonds (C3-C4, C5-C6), located in the “center” of the ring, exhibit the highest population (2.93e), and conversely, these bonds have the lowest population in the borabenzene ring (2.70e). Interestingly, the N-C bonds (N-C4, N-C5) are the least “saturated” with electron density (2.44e) compared to the other bonds in the NC_5_ ring (2.70e, 2.93e). In summary, the B and N atoms, with their different properties, contribute to distinct types of electron density concentrations with varying degrees of localization in the molecular rings.

The product of the reaction, the pyridine derivative of 1-borabarrelene (py→BBa), is a molecule that contains two double C=C bonds (C3=C4, C5=C6) in the C_5_H_5_B fragment, one double C=C bond (C1=C2) in the former acetylene unit, and two single C-C bonds (C1-C7, B-C2) formed between the C_5_H_5_B and HC≡CH molecules (see Scheme 4).

**Figure 7:**
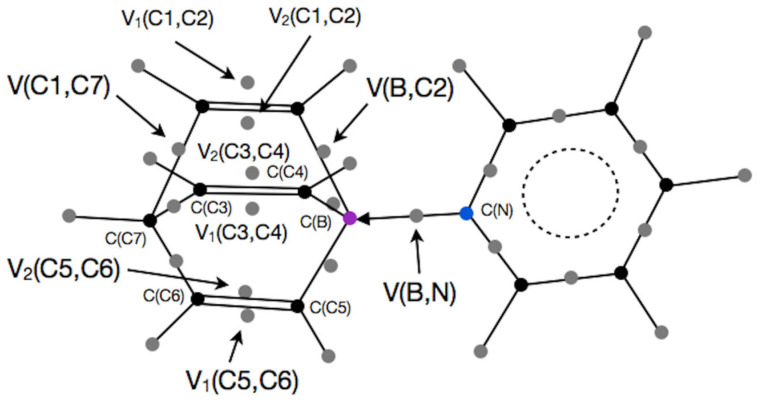
The core and valence attractors in the py→BBa molecule (pyridine derivative of 1-borabarrelene)—the product of the reaction. The analysis was performed using DFT(M062X) data.

The electronic structure is represented by 45 local maxima of the ELF, with 14 core and 31 valence attractors of the point type, as shown in Figure 7. Two new bonds, C1-C7 and B-C2, are represented by bonding disynaptic attractors V(C1,C7) and V(B,C2). The redistribution of electron density during the reaction leads to the formation of two new C=C bonds, C3=C4 and C5=C6, which are reflected by two pairs of bonding disynaptic attractors V_i=1,2_(C,C). This ELF topology, characteristic of a double C=C bond, was first described by Savin et al. [20]. Three pairs of V_i=1,2_(C,C) attractors are observed throughout the molecule, as the C1=C2 double bond also forms from the original HC≡CH fragment (see Figure 7).

**Figure 8:**
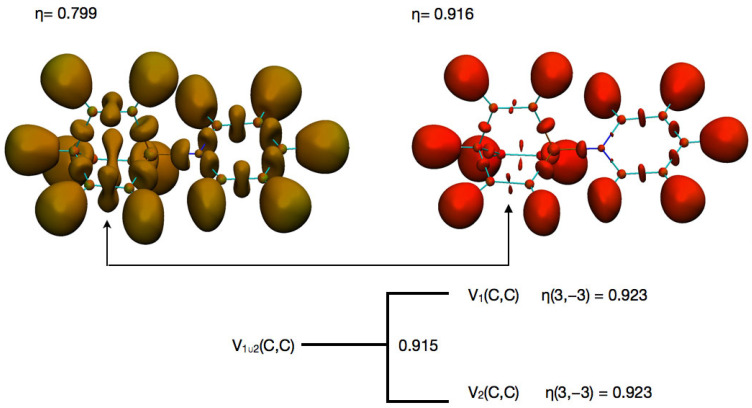
Distribution of ELF function values in the py→BBa molecule for η = 0.799 and 0.916. At η = 0.799, localization domains corresponding to chemical bonds are visible. For the three double bonds (C1=C2, C3=C4, and C5=C6), reducible domains encompass pairs of bonding attractors: V_i=1,2_(C1,C2), V_i=1,2_(C3,C4), and V_i=1,2_(C5,C6). At η = 0.916, which reflects higher electron localization, these appear as irreducible localization domains. Analysis based on DFT(M062X) data.

The localization domains are shown in Figure 8 for ELF values of 0.799 and 0.916. For each chemical bond, a localization domain is visible. At the smaller ELF value (0.799), each chemical bond is represented by a single localization domain. However, the detailed structure of the C=C double bonds is not clearly observed. In the regions of the C=C double bonds, a very high degree of electron localization is seen, with a separation between domains, which can be associated with the V_i=1,2_(C,C) basins that appear at an ELF value of 0.915. The corresponding bonding attractors V_i=1,2_(C,C) are found at 0.923. For the ELF value of 0.916, three pairs of small localization domains, representing the double C=C bonds, are clearly distinguishable.

The populations for the three pairs of V_i=1,2_(C1,C2), V_i=1,2_(C3,C4), and V_i=1,2_(C5,C6) basins corresponding to the C1=C2, C3=C4, and C5=C6 bonds are very similar, each with a population of 1.70e. Therefore, a total of 3.4e is localized for each C=C bond. The analysis of the single C-C and B-C bonds in the 1-borabicyclo [2.2.2]octa-2,5,7-triene fragment shows basin populations in the range of 1.92e–2.15e, typical for covalent-polarized (single) bonds. The B-C bonds, represented by the V(B,C) disynaptic basins, have populations between 2.09e and 2.15e, slightly higher than the C-C bonds, which range from 1.92e to 1.93e. The populations for the V_i=1,2_(C,C), V(C,C), and V(B,C) basins differ from the formal 4e and 2e values, with the “missing” electron density appearing in the H-C bonds, which have basin populations in the range of 2.08e–2.17e. The largest populations of 2.12e and 2.17e are calculated for the H-C bonds near the C=C bonds. A similar effect is observed for the pyridine ring. The basin populations for V(N,C) and V(C,C) are in the range of 2.47e–2.91e, with a total of 16.18e in the ring. The nitrogen-carbon bonds, with populations of 2.47e and 2.52e, are less saturated with electron density than the C^…^C bonds, which have basin populations ranging from 2.69e to 2.91e. The H-C bonds have populations greater than the formal 2e, ranging from 2.14e to 2.19e.

Analysis of the atomic contributions to the three B-C bonds (B-C2, B-C4, B-C5) reveals a dominance of the valence electron density from the C atoms, with values of 1.80e, 1.76e, and 1.76e, which account for 84% of the total population of the V(B,C) basins. The polarity index, p_CB_, is 0.68, indicating that the bonds are polarized towards the C atoms.

### 2.4. Reaction Mechanism—Joint Use of Topological Analysis of ELF and Catastrophe Theory

The mechanism of the py→BC_5_H_5_ + HC≡CH→py→BBa reaction, investigated using a combined topological analysis of the ELF function and CT, is presented as a sequence of ten successive structure stability domains (SSDs). The transitions between these SSDs are described by nine fold (F) catastrophes. Each SSD is defined by a specific set of ELF field attractors, which correspond to atomic cores, regions of non-bonding electron density, and chemical bonds. For simplicity, the terms structure stability domain and reaction phase will be used interchangeably throughout the discussion.

**Figure 9:**
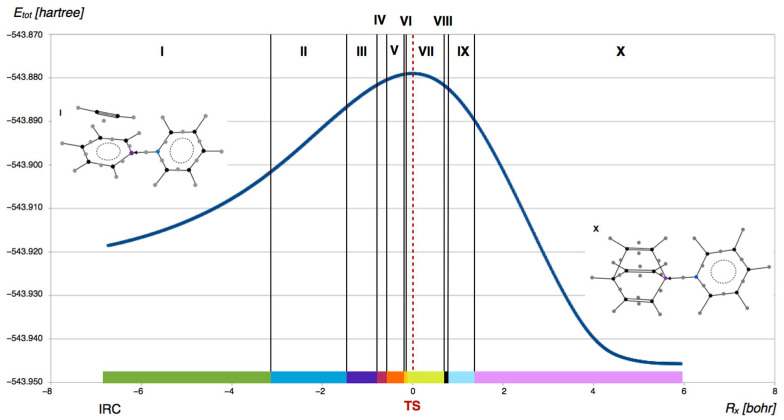
Graph illustrating the relationship between the total energy (E_tot_) of the reacting system and the IRC reaction coordinate (R_x_) with the structure stability domains marked. Each SSD corresponds to a qualitatively distinct electronic structure as determined by the topological analysis of the molecular ELF field. The barcode below the graph represents the phase lengths, defined by the number of points on the IRC path exhibiting the same ELF topology. The analysis uses DFT(M062X) data.

The curve of total energy (E_tot_) as a function of the IRC coordinate (R_x_), with the structure stability domains marked, is shown in Figure 9. As seen in the plot, the highest number of points occurs in phase I and the final phase X of the reaction. Notably, the transition state structure is not associated with any change in the topology of the ELF molecular field. However, as the system approaches the TS, the distances between individual ELF field catastrophes generally decrease.

**Figure 10:**
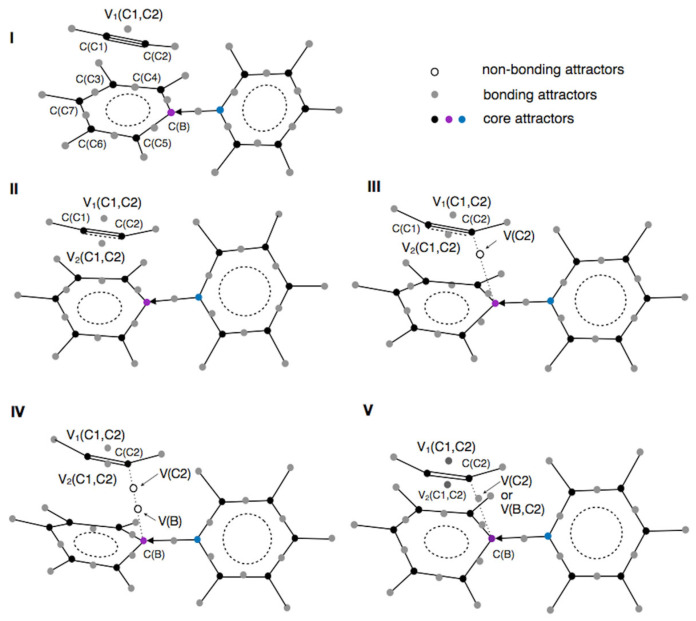
Core and valence attractors of the ELF field with Lewis formulas depicting the electronic structure in phases (structure stability domains) (**I**–**V**). The analysis was performed using DFT(M062X) data.

**Figure 11:**
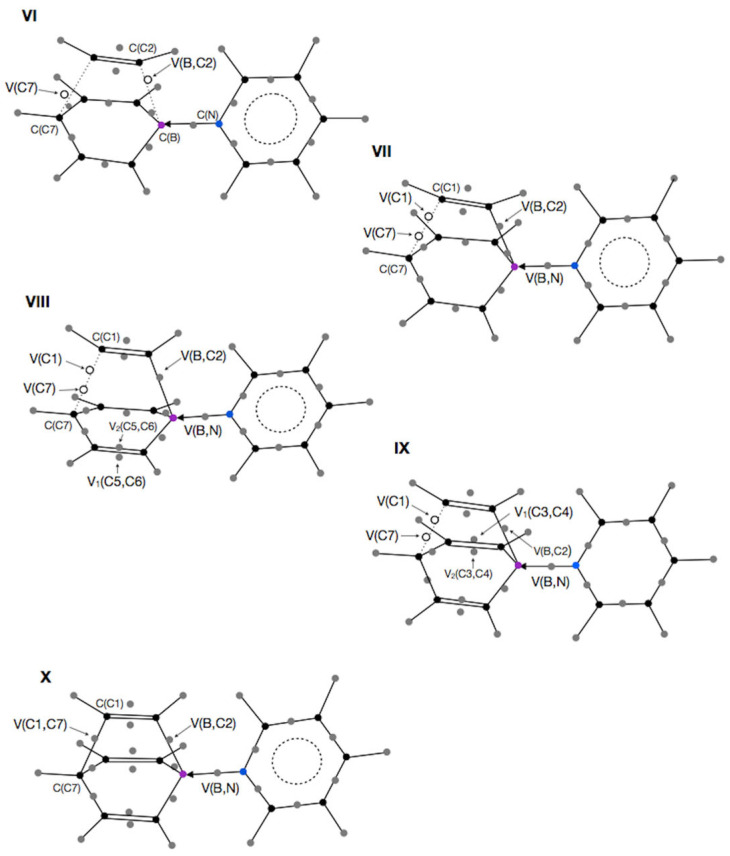
Core and valence attractors of the ELF field with Lewis formulas illustrating the electronic structure in phases (structure stability domains) (**VI**–**X**). The analysis using DFT(M062X) data. The B-C2 bond in phase VII is represented by a solid line due to the observation of the attractor V(B,C2) for most points in this SSD.

**Figure 12:**
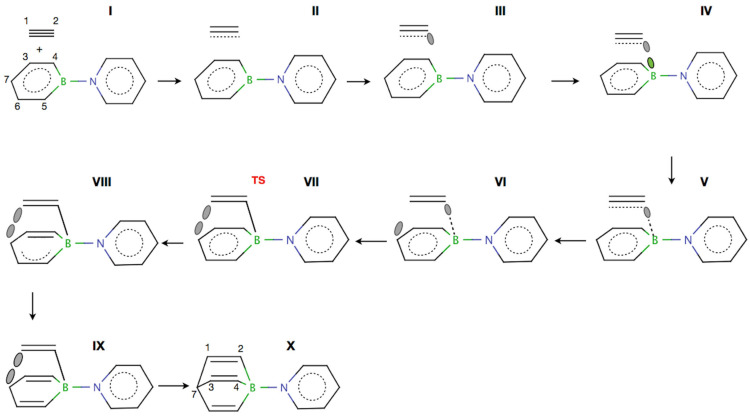
Modified Lewis formulas, adjusted according to the results of BET analysis, for the DA reaction between the pyridine adduct of borabenzene (py→BC_5_H_5_) and acetylene (HC≡CH), leading to the formation of the pyridine adduct of 1-borabarrelene (py→BBa). The B-C2 bond in phase VII is represented by a solid line due to the observation of the attractor V(B,C2) for most points in this SSD. These formulas illustrate the chemical bonds and their formation within each phase (structure stability domain) of the reaction. Analysis based on DFT (M062X) data.

The evolution of the ELF field attractors throughout the reaction is shown in Figure 10 (phases I–V) and Figure 11 (phases VI–X). To complement this, the modified Lewis structures in Figure 12 illustrate the changes in the electronic structure of the reacting molecules in a way that should be accessible to a broad audience of chemists. The representation code includes standard bond types—single (-), double (=), and triple (≡) bonds—as well as bonds of intermediate character between double and triple bonds, delocalized π systems (^…^), and a graphical element in the form of colored ellipses. These ellipses represent non-bonding localization basins, V(C) and V(B), which appear or disappear as a result of the formation or cleavage of the C1-C7 and B-C2 bonds. Such basins are identified in phases III, IV, VI, VII, VIII, and IX.

Below, the topology of the ELF function will be discussed for each SSD, accompanied by a chemical interpretation focused primarily on the formation and breaking of chemical bonds. The values of the average electron population for localization basins at the first point of the IRC path corresponding to each reaction phase are summarized in Table 3. The atom numbering, and consequently the labeling of ELF attractors and localization basins, is provided in Figure 10 and Figure 11. In the following description of the reaction mechanism, the attractors and basins corresponding to the C1≡C2 bond in HC≡CH and the C3^…^C4 and C5^…^C6 bonds in py→BC_5_H_5_ are denoted as V_1_(C1,C2), V_1_(C3,C4), and V_1_(C5,C6), respectively.

#### 2.4.1. Phase I

At the very early stage of the reaction, the reacting system consists of a py→BC_5_H_5_⋯HC≡CH molecular complex with weakly interacting molecules. Both the py→BC_5_H_5_ and HC≡CH molecules retain electronic structures similar to those observed for the isolated molecules at their total energy minima. The weak interactions between the molecules do not alter the topology of the ELF in either subunit (see Figure 10). The total number of core and valence attractors is 40, and this phase persists for 139 points along the IRC path.

In the MC, the HC≡CH molecule possesses three valence disynaptic attractors in the valence shell: one bonding disynaptic attractor, V_1_(C1,C2), and two protonated disynaptic attractors, V(H,C1) and V(H,C2). The V_1_(C1,C2) attractor and basin describe the electron density of the covalent carbon-carbon bond, which is formally triple.

The ELF topology described in this phase is maintained until the C1 and C2 atoms from the HC≡CH molecule approach the C7 and B atoms in the py→BC_5_H_5_ molecule, with r(C1,C7) and r(C2,B) distances equal to 2.71 Å and 2.62 Å, respectively.

During the course of the reaction, the total basin population for the carbon-carbon bond in HC≡CH increases from 5.17e in the isolated acetylene molecule to 5.25e at the first point on the IRC path (R_x_ = −6.38 bohr), and finally to 5.22e at the last point of phase I. The total energy difference between the first and last points on the IRC path in this phase is 10.06 kcal/mol.

#### 2.4.2. Phase II

In the second phase of the reaction, which is defined for 71 points on the IRC path, both molecules remain separate chemical entities. However, the mutual interaction between the molecules and the redistribution of electron density leads to the appearance of a second valence attractor, V_2_(C1,C2), of point type in the region of the C≡C bond in HC≡CH. The first catastrophe of the ELF field is a fold (F^+^) and occurs around the r(C1,C7) and r(C2,B) distances, which are 2.71 and 2.62 Å, respectively. Two new critical points (CPs) of indices 0 and 1 appear, with a distance of 0.16 Å between them. The total number of core and valence attractors increases to 41.

From an ELF topological perspective, the C≡C bond in the HC≡CH molecule now resembles the C=C bond in the ethylene (C_2_H_4_) molecule. In the energy minimum of C_2_H_4_, two attractors and basins, V_i=1,2_(C,C), are observed for the C=C bond [20]. This change in ELF topology is expected because the final product of the reaction contains an alkene -C=C- group. The ELF topology in the py→BC_5_H_5_ remains unchanged compared to the isolated molecule, indicating that no disturbance of its electronic structure occurs.

Phase II continues until the r(C1,C7) and r(C2,B) distances reach values of 2.48 Å and 2.39 Å, respectively. Throughout the reaction, the total basin population for the carbon-carbon bond in HC≡CH decreases slightly from 5.22e to 5.18e. Examining the populations of the V_1_(C,C) and V_2_(C,C) basins, an equalization of their populations is observed. At the start of the phase, their populations were 1.80e and 3.42e, respectively, while at the end of the phase, the values were calculated as 2.55e and 2.63e.

The total energy difference between the first and last point on the IRC path in this phase is 9.20 kcal/mol.

#### 2.4.3. Phase III

Phase III continues for 29 points along the IRC path. The number of core and valence attractors increases to 42. This increase, compared to the previous phase (phase II), results from the emergence of the V(C2) monosynaptic attractor and basin in the acetylene molecule (see Figure 10). The corresponding catastrophe is of the fold (F^+^) type and is observed at approximately r(C1,C7) = 2.48 Å and r(C2,B) = 2.39 Å.

Notably, the electron density concentrated in the C2···B region undergoes a change in its localization properties: the local ELF maximum is now observed closer to the C2 atom rather than the B atom. The increased probability of electron density localization in the valence region of carbon may result from the rehybridization of the occupied p orbitals in the C≡C fragment, which participate in B-C bond formation.

At the beginning of phase III, the populations of the V_1_(C1,C2) and V_2_(C1,C2) basins in the HC≡CH molecule are 2.48e and 2.57e, respectively, totaling 5.05e. By the end of the phase, these values decrease to 2.42e and 2.36e, respectively, with a total population of 4.78e. Meanwhile, the V(C2) monosynaptic basin shows a gradual increase in electron density, with its population rising from 0.14e at the beginning of the phase to 0.46e at the end point of the phase.

During this phase, the total energy increases by 3.09 kcal/mol, while the r(C1,C7) and r(C2,B) distances reach 2.38 Å and 2.29 Å, respectively. At these distances, another catastrophe in the ELF field is observed, marking a topological change in the molecular ELF field that defines the next phase of the reaction.

#### 2.4.4. Phase IV

The mentioned catastrophe is the third fold (F^+^) type observed since the beginning of the reaction. It occurs in the vicinity of the B atom within the py→BC_5_H_5_ molecule. The total number of attractors at this point is 43. In the valence shell of boron, a new attractor, a corresponding basin, and a critical point of index 1 emerge. The newly formed V(B) basin is classified as non-bonding and monosynaptic. Its population begins at 0.01 e at the start of this phase and increases slightly to 0.03 e by the final point. It is worth emphasizing how negligible this population is, which may raise interpretational challenges from a chemical perspective. The low population of the V(B) basin appears to correlate with the limited contribution of the B atom’s electron density to the formation of boron–carbon bonds. In contrast, the population of the V(C2) basin increases from 0.48 e to 0.59 e, indicating a continuous “saturation” process of this basin. From a chemical point of view, the formation of the B-C2 bond remains in progress.

Phase IV spans only 9 points along the IRC path. During this phase, both the r(C1,C7) and r(C2,B) distances decrease by approximately 0.05 Å, while the total energy increases by 0.64 kcal/mol. The final point of this phase is reached at distances of 2.35 Å for C1-C7 and 2.26 Å for C2–B.

#### 2.4.5. Phase V

The electronic structure in phase V is determined by a fold catastrophe (F), during which the monosynaptic, nonbonding attractor and basin, V(B), in the py→BC_5_H_5_ molecule are annihilated. The V(B) attractor and its adjacent saddle point coalesce. For the point on the IRC path preceding the fold, the distance between critical points with indices 0 and 1 was 0.04 Å, with ELF values of 0.53. As a result, the total number of attractors decreases to 42. The attractor and basin V(C2) remain unchanged throughout this catastrophe. The basin population for V(C2) starts at 0.65e at the beginning of the phase and increases to 0.88e at the final point.

The annihilation of the V(B) attractor poses a challenge in providing a clear chemical interpretation for the observed ELF topology change. The very small (<0.03e) value of the V(B) basin electron population and the high degree of delocalization of the electron density indicate that the contribution of electrons from the B atom valence shell in the process of boron-carbon bond formation is negligible. The bond is primarily formed by electron density transfer from the C atom valence shell, which is reflected by the formation of the V(C2) nonbonding basin in the C2⋯B region in phase III.

Analyzing the topology of the ELF field in the C2⋯B bonding region in the subsequent phases (see Figure 10 and Figure 11), one can notice the lack of qualitative changes between the phases. The V(C2) attractor remains unchanged, while the population of its basin gradually increases. At the beginning of phase III, the V(C2) attractor is located at a distance of 0.73 Å from the C(C2) attractor, while in the reaction product, it is at a very similar distance of 0.78 Å. This shows that, during the approach of the reacting HC≡CH and py→BC_5_H_5_ molecules, the position of the V(C2) attractor in relation to the position of the carbon atom core, C(C2), changes only slightly. The fraction of the distance r(C2,B) at which the V(C2) attractor is located at the beginning of phase III is 0.31, and in the reaction product, it is 0.48. After the reaction is complete, the attractor V(C2) is located near the bond center, which is typical for the ELF topological description of a covalent bond. These observations allow us to hypothesize that the final stage of the B-C2 bond formation begins in phase V.

There remains the question of the synapticity of the V(C2) basin. In the reaction product, the V(B,C2) attractor and basin are observed, so from a certain distance, r(C2,B), we should expect a change in synapticity from the monosynaptic (non-bonding) V(C2) type to the disynaptic (bonding) V(B,C2). Further studies show that the change in synapticity occurs in phase VII, and a detailed description is presented in the “Discussion” section.

The total population for the two V_1_(C1,C2) and V_2_(C1,C2) basins in HC≡CH is 4.66e at the beginning of the phase, and it decreases to 4.49e at the last point. Due to the population being close to the formal value of 4e, we can speak of a C=C double bond.

This phase lasts for 17 points along the IRC path, with the reaction coordinate changing from −0.56 bohr to −0.19 bohr. The total energy increases by 0.79 kcal/mol. The r(C1,C7) and r(C2,B) distances decrease by approximately 0.11 Å, and the final point is observed at distances of 2.29 Å and 2.21 Å, respectively.

#### 2.4.6. Phase VI

Considering the number of points on the IRC path, phase VI is extremely short. Only one point has been identified at approximately −0.16 bohr (R_x_), with the topology of the ELF function characterized by 43 core and valence attractors (see Figure 11). The fold (F^+^) catastrophe, observed at approximately r(C1,C7) and r(C2,B) distances of 2.29 Å and 2.20 Å, leads to the appearance of a new attractor, basin V(C7), and a critical point of index 1 in the vicinity of the carbon C7 atom in the py→BC_5_H_5_ molecule. From both a structural and ELF topological perspective, the process of forming the second bond (C1-C7) between the reacting molecules has begun. To introduce a pictorial analogy to the construction of a bridge, in which initially two bridge abutments are built, in the phase discussed, the construction of the “abutment” on the C7 side has been initiated. The new attractor and basin, V(C7), belong to the non-bonding and monosynaptic types. Interestingly, the formation of the second intermolecular bond (C1-C7, following the B-C2 bond) begins in the py→BC_5_H_5_, whereas the B-C2 bond was initiated by a significant concentration of electron density and a change in electron localization properties at the C2 atom in the HC≡CH molecule.

The population of the V(C7) basin is 0.12e, while that of the V(C2) basin, which is in a continuous process of “saturation” with electron density, is 0.89e. In the two bonding basins, V_1_(C1,C2) and V_2_(C1,C2) in the HC≡CH molecule, 4.47e is concentrated, which allows us to interpret the C1=C2 bond as being close to a double bond. The total energy increases by approximately 0.02 kcal/mol.

#### 2.4.7. Phase VII

The next catastrophe occurs at r(C1,C7) and r(C2,B) distances of 2.28 Å and 2.20 Å, respectively. This catastrophe is a fold (F^+^) occurring near the C1 carbon core of the acetylene fragment, where a new attractor, basin, and critical point of index 1 appear (see Figure 11). The new attractor and basin, V(C1), are of the non-bonding and monosynaptic type. Using the previously mentioned analogy of building a bridge, the construction of a second “abutment” on the C1 site of the HC≡CH molecule has been initiated.

The topological analysis of the ELF reveals 44 core and valence attractors in the reacting system (see Figure 11). As shown in Figure 9, phase VII also includes the transition state structure of the reaction, where the r(C1,C7) and r(C2,B) distances are 2.266 Å and 2.175 Å, respectively.

For the transition state structure, in the acetylene fragment, there are two localized bonding disynaptic attractors, V_i=1,2_(C1,C2), for the carbon-carbon bond, and two protonated disynaptic attractors, V(H,C), corresponding to the H-C bonds. The basin populations of 2.21e and 2.29e for the V_i=1,2_(C1,C2) basins, totaling 4.5e, suggest the presence of a double C1=C2 bond. From the first point studied on the IRC path, where the population of V(C1,C2) was 5.25e, a significant depopulation of the primarily triple bond is observed.

The two six-membered rings of the py→BC_5_H_5_ molecule are characterized by two sets of six bonding disynaptic attractors: V(C,C), V(B,C), and V(N,C), which lie approximately in the planes of these rings. The C-C bonds in the NC_5_ ring have basin populations ranging from 2.49e to 3.04e, while those in the BC_5_ ring range from 2.46e to 2.91e. The B-C bonds exhibit similar $\bar{N}$ values of 2.51e and 2.55e. The N-C bonds, with basin populations of 2.46e and 2.47e, are single bonds.

The analysis of the two C-C bonds in the BC_5_H_5_ fragment, which will acquire double bond character (C=C) by the end of the reaction, shows similar basin populations of 3.04e. From the first point of the reaction, their populations (2.72e, 2.74e) increased by about 0.3e. Bonds with populations close to 3e cannot be classified as single bonds, but are rather intermediate between single and double bonds. This interpretation aligns with the expected aromatic character of the analyzed rings.

**Figure 13:**
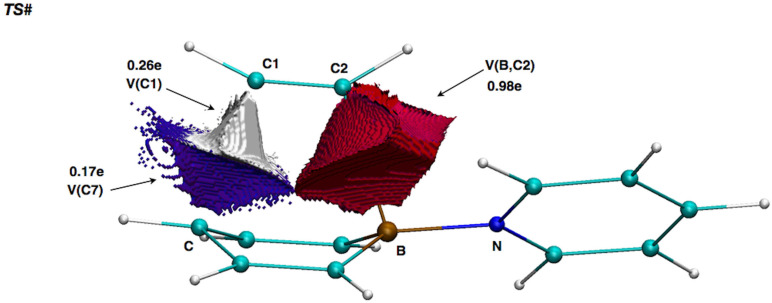
V(C1), V(C7) localization basins for the C1-C7 bond and V(B,C2) localization basin for the B-C2 bond with the corresponding values of the basin population in the transition state structure of the py→BC_5_H_5_ + HC≡CH→py→BBa reaction. The analysis using DFT(M062X) data.

The most interesting regions, C1⋯C7 and C2⋯B, where new bonds are continuously forming, are characterized by two monosynaptic nonbonding basins, V(C1) and V(C7), and a single bonding disynaptic basin, V(B,C2), respectively (see Figure 13). The C1-C7 bond between the acetylene and the py→BC_5_H_5_ fragments is not yet fully formed, as evidenced by the basin populations of V(C1) and V(C7), which are 0.17e and 0.26e, respectively. The larger value is associated with the V(C1) basin localized near the carbon core of the HC≡CH fragment. The boron-carbon bond, with a basin population of 0.98e, can be considered as partially formed. However, it is still in the process of formation, as its population is significantly below the approximately 2e expected for a fully established single bond.

The topology of the ELF described above has been observed for a total of 37 points along the IRC path. Of these, 6 points were identified before the TS, and 31 points were recorded as the system traversed the TS (see Figure 9). During this phase, the r(C1,C7) and r(C2,B) distances were reduced by approximately 0.24 Å and 0.23 Å, respectively. The saturation of the B-C2 bond and the C1⋯C7 region (V(C1) and V(C7) basins with electron density is clearly evident. The basin population of V(B,C2) increases from 0.91e (V(C)) to 1.35e, while the total population of the two V(C) basins rises from 0.35e to 0.84e. The last point in this phase was identified at r(C1,C7) and r(C2,B) distances of 2.16 Å and 2.08 Å, respectively.

The reaction coordinate for phase VII changes from −0.14 bohr to 0.71 bohr. The total energy on the way to TS increases by 0.06 kcal/mol and then decreases by 1.87 kcal/mol.

#### 2.4.8. Phase VIII

Phase VIII has been identified at only two points on the IRC path, and is immediately followed by the next fold (F^+^) catastrophe, which determines the topology of the ELF function in phase IX. The chemical significance of fold catastrophes leading to phase VIII and IX is very similar: both lead to the formation of localized double C=C bonds from C^…^C bonds in the BC_5_H_5_ fragment (see Figure 11 and Figure 12).

From the ELF topological perspective, there are now two double C1=C2 and C3=C4 bonds already present in the reacting system. Interestingly, the formation of the double C3=C4 bond occurs before the final establishment of the C1-C7 bond. However, it is important to note that until the energy minimum of the reacting system is reached, no bond can be considered fully formed, as electron density continuously “flows.” The fold catastrophe in this step results in the formation of the second disynaptic attractor and basin V_2_(C3,C4), along with a critical point of index 1. The catastrophe occurs for the r(C3,C4) distance of 1.35 Å. In the region of the C3=C4 bond, two bonding disynaptic attractors and basins V_1_(C3,C4) and V_2_(C3,C4) are found. The number of core and valence attractors increases by 1, reaching 45.

The basin populations for V_1_(C3,C4) and V_2_(C3,C4) are 1.50 and 1.64e, respectively, after the fold. The population of the boron-carbon bond represented by the V(B,C2) basin is 1.36e, while that of the V(C1) and V(C7) basins, which are in a continuous process of “saturation” with electron density, is 0.49e and 0.36e, respectively. In the two bonding basins V_1_(C3,C4) and V_2_(C3,C4) in the HC≡CH molecule, 2.05 and 2.04e are “concentrated”, which allows us to interpret the C1=C2 bond as a double bond (4.09e in total).

The last point in this phase is found for r(C1,C7) and r(C2,B) distances of 2.15 Å and 2.07 Å, respectively. The total energy decreases by about 0.13 kcal/mol.

#### 2.4.9. Phase IX

Phase IX is characterized by 27 points on the IRC path, all exhibiting the same ELF topology, defined by 46 core and valence attractors. The total energy decreases by 4.94 kcal/mol during this phase. The increase in attractor count, compared to phase VIII, is due to the second fold (F^+^) catastrophe occurring in the region of the C5-C6 bond of the BC_5_H_5_ fragment. The catastrophe is observed for the r(C5,C6) distance of 1.35 Å. A single disynaptic attractor and basin V_2_(C5,C6) emerge. The populations for V_1_(C5,C6) and V_2_(C5,C6) following the catastrophe are 1.52e and 1.63e, respectively, resulting in a total of 3.15e. These values closely match the populations of the V_1_(C3,C4) and V_2_(C3,C4) basins formed during the fold in phase VIII, when the first C3^…^C4 bond was “transformed” into a double C3=C4 bond. From the ELF topological perspective, all three double C=C bonds in the BC_5_ ring, predicted by the theory of DA reactions, are already established, though they are not yet fully “saturated” with electron density. Notably, the formation of the second double C3=C4 bond in the BC_5_H_5_ fragment precedes the closure of the ring involving the former acetylene unit (C1-C7 bond).

The final point on the IRC path for phase IX corresponds to the r(C1,C7) and r(C2,B) distances of 2.051 Å and 1.988 Å, respectively. The $\bar{N}$ value of the C5=C6 bond increases to 3.22e (1.56e, 1.66e) in this phase. A similar effect is observed for the population of the C3=C4 bond, which increases from 3.15e (1.49e, 1.66e) to 3.21e (1.54e, 1.67e). The populations of the V(C1) and V(C7) basins increase from 0.50e and 0.38e (0.88e in total) to 0.63e and 0.50e (1.13e in total). In the case of the two bonding basins V_1_(C1,C2) and V_2_(C1,C2) in the HC≡CH molecule, a new effect is observed, as the total population of both basins slightly decreases from 2.04e and 2.04e (4.08e in total) to 1.95e and 1.95e (3.90e in total). Nevertheless, the topological analysis of ELF shows that the C1=C2 bond still exhibits double bond character.

#### 2.4.10. Phase X

**Figure 14:**
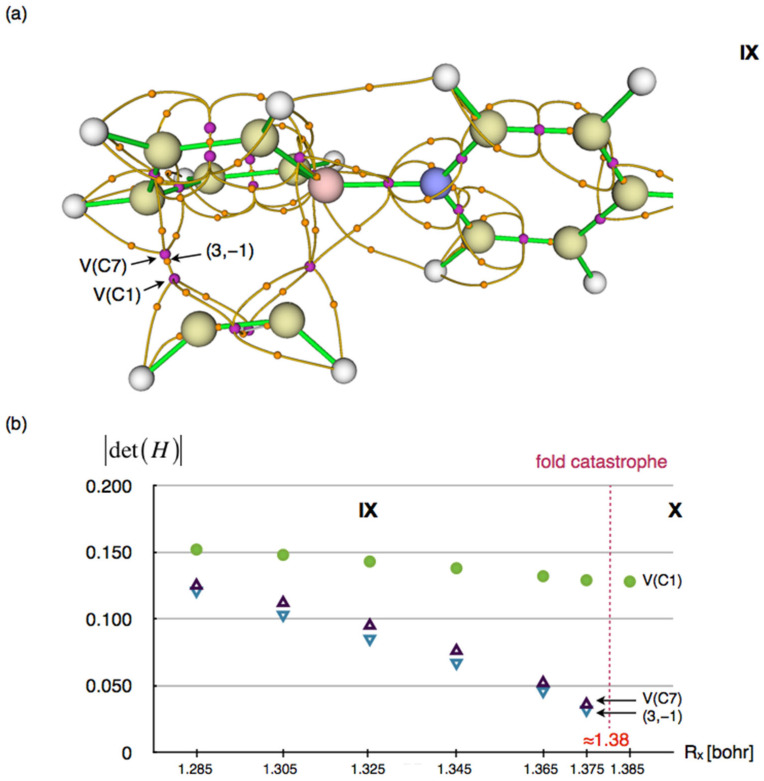
(**a**) Critical points of the ELF field with indices 0 (attractors) and 1 (3,–1), shown together with gradient paths and the geometric structure of the reacting system in phase IX, for the reaction coordinate R_x_ = 1.293 bohr. (**b**) Evolution of the absolute value of the Hessian matrix determinant, |det(H)|, for critical points describing the change in ELF field topology in the C1⋯C7 interatomic region, leading to the formation of the C1-C7 bond. In the fold (F) catastrophe, the attractor V(C7) and the nearby critical point (3,–1) undergo coalescence. Analysis based on DFT (M062X) data.

The topology of the ELF function in the final phase X is determined by a fold catastrophe (F) in which the attractor and basin V(C7) and the critical point of index 1 are annihilated. ELF topology analysis performed for the last point on the IRC path preceding the fold showed that the critical points with index 0 (attractor V(C7)) and index 1, which will coalesce, are located at a distance of 0.015 Å, with ELF values of 0.807 and 0.807, respectively. In the C1⋯C7 region, only a single attractor and basin, V(C1,C7), are now localized. A detailed illustration of the location of the critical points in the C1⋯C7 region involved in the catastrophe is shown in Figure 14a.

The analysis of changes in the absolute values of the Hessian matrix determinants, |det(H)|, calculated for the attractors V(C1), V(C7), and the critical point of index 1 located between them—at points along the IRC reaction path in phase IX preceding the fold (see Figure 14b)—shows that only the attractor V(C7) and the nearby index 1 critical point undergo coalescence, as indicated by the convergence of their |det(H)| values toward zero. In contrast, the change in |det(H)| for the attractor V(C1) during phase IX is minimal and does not show any tendency to approach zero. After the fold, in phase X, the value of |det(H)| for V(C1) remains close to that calculated at the endpoint of phase IX.

Considering the small separation between the attractor V(C7) and the CP of index 1 (see Figure 14a), along with the observed evolution of the |det(H)| values for the CPs (see Figure 14b), the qualitative change in the topology of the molecular ELF field in the C1⋯C7 region near the reaction coordinate R_x_ ≈ 1.38 bohr can be characterized as the simplest fold catastrophe. From an ELF topological point of view, the C1-C7 bond is formed. Likewise, the reaction can be considered complete, as no further changes in ELF topology are observed. However, the process of “saturation” of the localization basins will continue until the molecule reaches its total energy minimum.

The total number of attractors decreases to 45. Phase X is identified over 192 points along the IRC path, with the reaction coordinate ranging from 1.40 bohr to 5.36 bohr (the final point obtained in the calculations).

After the fold, the basin population of V(C1,C7) is 1.15e. The three double C=C bonds, represented by the V_i=1,2_(C,C) pairs, have populations of 3.21e (1.54e, 1.67e), 3.21e (1.55e, 1.66e), and 3.88e (1.94e, 1.94e), respectively. The C1=C2 bond in the former acetylene fragment stands out due to its relatively high population compared to the newly formed C3=C4 and C5=C6 bonds in the BC_5_ ring. The B-C2 bond, represented by the V(B,C2) basin, has a population of 1.59e. The electronic structure of the pyridine derivative of 1-borabarrelene is discussed in detail in Section 2.3.

Phase X represents the final stage of the reaction between the HC≡CH and py→BC_5_H_5_ molecules in which a change in the topology of the ELF molecular field is observed.

To sum up, the reaction between HC≡CH and py→BC_5_H_5_ proceeds through ten phases and involves the following: (1) Redistribution and changes in the localization properties of electron density within the C≡C bond of the HC≡CH molecule, as well as the neighboring C2⋯B and C1⋯C7 regions. The reorganization of the electron density in the C1=C2 region ultimately leads to the formation of a C1=C2 double bond; (2) concentration of electron density in the C2⋯B region, where a single B-C2 bond will form as a result of a qualitative change in electron localization properties; (3) concentration of electron density in the C1⋯C7 region, where a single C1-C7 bond will form as a result of a similar qualitative change in electron localization properties; and (4) redistribution of electron density in the regions of the C3⋯C4 and C5⋯C6 bonds and their neighboring bonds, leading to the localization of pairs of local ELF field attractors and the formation of localized C3=C4 and C5=C6 double bonds.

## 3. Discussion

**Figure 15:**
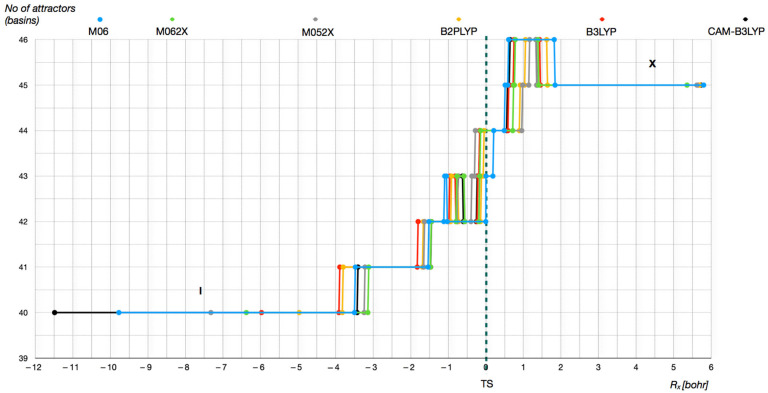
Number of core and valence (monosynaptic and disynaptic) attractors and basins in the reacting py-BC_5_H_5_ and HC≡CH system in the individual reaction phases (I–X) and depending on the electron density functional used. The analysis using DFT(M062X) data.

The mechanism of the reaction between the pyridine adduct of borabenzene, py→BC_5_H_5_, and acetylene, HC≡CH (see Scheme 4), has been elucidated using a combined topological analysis of ELF and CT. Six electron density functionals were employed to assess the potential influence of the theoretical method on the BET-based picture of the reaction mechanism. A graph illustrating changes in the number of attractors and basins in the molecular ELF field across different reaction phases, as determined by each electron density functional, is presented in Figure 15.

As shown by the detailed analysis of the results obtained using the DFT(M062X) method, the reaction proceeds through ten distinct phases, each characterized by the formation or annihilation of ELF attractors and basins. The same number of phases was identified using the remaining electron density functionals. The Lewis structures, modified according to the ELF topological analysis to reflect bond formation during each reaction phase, are presented in Figure 12.

The total number of attractors and localization basins changes from 40—identified for the weakly interacting py→BC_5_H_5_ and HC≡CH reactants in phase I—to 46 in the penultimate phase IX. As the reaction progresses and the system approaches the transition state structure, the number of attractors and basins generally increases (see Figure 15). A decrease in attractor count is observed during transitions to phases where the formation of the intermolecular B-C2 bond is associated with the annihilation of the V(B) attractor, and where the formation of the C1-C7 bond coincides with the annihilation of the V(C7) attractor.

Interestingly, the highest number of attractors does not correspond to the TS (or its associated phase), but rather occurs in phase IX, after the TS has been passed. In this phase, each of the C=C double bonds in the BC_5_ ring are characterized by a pair of attractors: V_i=1,2_(C3,C4), V_i=1,2_(C5,C6), and the intermolecular C1-C7 bond is in the final stage of formation (with the V(C1) and V(C7) basins still present).

The number of core and valence attractors across phases I to X using DFT(M062X) data is as follows: 40 (I), 41, 42, 43, 42, 43, 44 (TS), 45, 46, and 45 (X).

An analysis of the types and sequence of ELF field catastrophes observed during the reaction reveals a catastrophe sequence—originally introduced in 2003 [35]—as follows: HC≡CH + py→BC_5_H_5_: 1-10-F^+^F^+^F^+^FF^+^F^+TS^F^+^F^+^F-0: py→BBa. All catastrophes identified are of the simplest type, fold (F), and none occur simultaneously with another.

**Figure 16:**
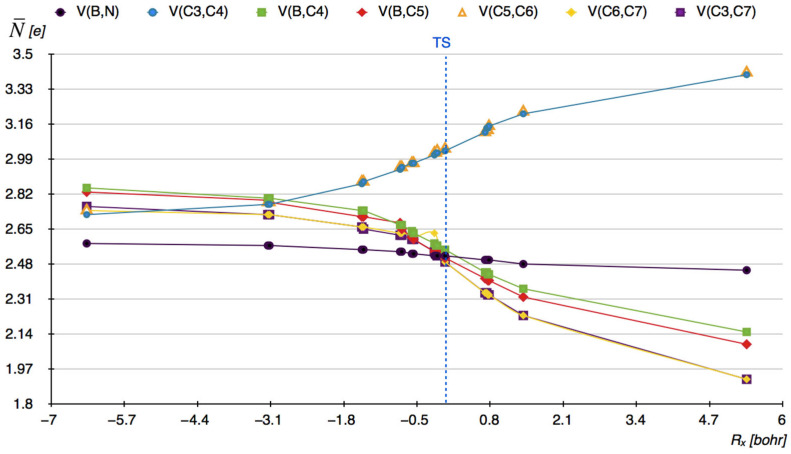
Evolution of the electron populations (in e) of selected localization basins during the course of the py→BC_5_H_5_ + HC≡CH→py→BBa reaction. Values for initial points with coordinates Rx (in bohr) in phases I to X are presented. Lines connecting the points are added to highlight the trends of changes. The analysis is using DFT(M062X) data.

As shown by the BET analysis, the reaction between the py→BC_5_H_5_ and HC≡CH molecules involves a significant redistribution of electron density—primarily from the region of the formally triple C1≡C2 bond in acetylene and B^…^C4, B^…^C5, C3^…^C7, and C6^…^C7 bonds in the BC_5_ ring to the C2⋯B, C1⋯C7 regions and the C3^…^C4 and C5^…^C6 bonds, which in the py→BBa product acquire the character of localized double bonds (C3=C4 and C5=C6). Figure 16 illustrates the evolution of the population of localization basins describing chemical bonds in the interacting molecules for the initial point of each phase along the IRC path. The populations of the V(C3,C4) and V(C5,C6) basins gradually increase from 2.72e and 2.74e to 3.40e and 3.40e, respectively, between the reactant and the product. In contrast, the populations of basins representing the other bonds in the BC_5_ ring—namely V(B,C4), V(B,C5), V(C6,C7), and V(C3,C7)—gradually decrease. Their populations drop from initial values of 2.83e, 2.85e, 2.74e, and 2.76e to final values of 2.15e, 2.09e, 1.92e, and 1.93e, respectively. This redistribution reflects a change in topological bond order from approximately 1.4 to around 1, confirming the transformation of initially delocalized B^…^C and C^…^C ring bonds into localized single B-C and C-C bonds. The magnitude of these changes can be compared with that observed for the B-N bond, whose population decreases only slightly over the course of the reaction—from 2.58e to 2.44e (see Figure 16).

The change in the localization properties of electron density within the C≡C bond in the acetylene molecule (phase II) leads to the formation of the second valence attractor, V_2_(C1,C2). The ELF topology, characterized by two local attractors, V_i=1,2_(C1,C2), is typical for a C=C double bond [20]. From an ELF topological point of view, the C≡C bond in acetylene already exhibits features of a double bond. However, the total basin population for V_i=1,2_(C1,C2), which is 1.80e and 3.44e (total 5.22e), still indicates a triple bond character. This is evident when comparing this value to the population of the V(C,C) basin in the isolated HC≡CH molecule, which is 5.15e, and at the beginning of phase I, where it is 5.25e. Analysis of further points along the IRC path shows that the total basin population gradually decreases, with the following values at the beginning of the subsequent phases I-X: 5.25e (I), 5.22e (II), 5.05e (III), 4.77e (IV), 4.66e (V), 4.47e (VI), 4.46e (VII), 4.09e (VIII), 4.08e (IX), and 3.88e (X). For the py→BBa molecule (at the energy minimum), the population of the two V_i=1,2_(C1,C2) basins is 1.70e and 1.70e (3.40e), which suggests a decrease in the double bond character of the C=C bond due to the participation of the single C-C bond in the equilibrium of resonance structures. Thus, assuming somewhat arbitrarily that the formal notation of the C=C double bond can be applied to basin populations with values between 4.5e and 3.5e, we can consider the C1=C2 bond to have a double bond character in the reacting system only starting from phase VI.

The “construction” of the B-C2 bond begins in phase III with a change in the topology of the ELF field in the valence region of the C2 atom in HC≡CH, which leads to the formation of a monosynaptic, nonbonding attractor and basin, V(C2). In the next phase, IV, a similar monosynaptic, nonbonding attractor and basin, V(B), is formed in the valence region of the B atom. In phase V, V(B) undergoes annihilation, and the synapticity of the basin V(C2) changes to V(B,C2), followed by the “saturation” of the basin V(B,C2) with electron density, which continues up to the last point on the IRC path. At the beginning of the final X phase, the population of V(B,C2) is 1.59e (see Table 3), while in the py→BBa molecule optimized for minimum energy, it is 2.15e, which corresponds to a typical B-C single bond.

The carbon-carbon bond (C1-C7) is formed as a result of three ELF field catastrophes, which determine the topology of the molecular ELF field in phases VI, VII, and the final X. In phase VI, an attractor and a nonbonding monosynaptic basin, V(C7), are formed in the valence region of the C7 atom in the py→BC_5_H_5_ fragment. In phase VII, an analogous attractor and basin appear in the valence region of the C1 atom in the HC≡CH fragment. Finally, in phase X, instead of two attractors and basins, V(C1) and V(C7), only one attractor and basin, V(C1,C7), appears after the annihilation of V(C7).

It is worth noting that in the case of B-C2 bond formation, the attractor and basin V(C2) are formed first in the HC≡CH molecule, whereas in the C1-C7 bond formation process, the attractor and basin V(C7) are created first in the py→BC_5_H_5_ molecule. It seems reasonable to associate this order of formation of the non-bonding V(C2) and V(B) attractors with the nature of the B-C2 bond, which is dominated by electron density from the C atom. In the isolated borabenzene molecule, analysis of the quantum atom contributions shows that the two B-C bonds forming the BC_5_ ring, with a population of 2.87e, consist of 17% electron density from the B atom. Similarly, in the py→BBa molecule, the three B-C bonds, formally single, with populations of 2.09, 2.09, and 2.14e, consist of 16% electron density from the B atom. Thus, the dominant contribution of electron density from the C atom seems to be reflected in the creation of the attractor and basin V(C2) first (phase III). In turn, the small contribution of electron density from the B atom is reflected in the creation of the attractor and basin V(B) later in phase IV.

Although, from a formal point of view (distinction between covalent-polarized and donor–acceptor bonds proposed by Krokidis et al. [17]), the B-C bond has a covalent-polarized character, it is easy to notice that the very small contribution of electron density from the B atom may suggest some donor–acceptor character of the bond. This interpretation is also supported by the observation that the population of the V(B) basin, from the moment of its creation (R_x_ ≈ −0.77 bohr) to the moment of its annihilation (R_x_ ≈ −0.59 bohr), is negligible and changes in the range of 0.01e–0.03e (phase IV). Regardless of the very small population value, it should be noted that all computational methods used in the discussed studies show the presence of the attractor and basin V(B) (see Figure 15), which suggests that, although it is not a computational artifact, its chemical interpretation remains problematic. In summary, the analysis of the ELF field attractors in the B⋯C2 region and their evolution during the reaction confirms that the B-C bond has a covalent-polarized character.

**Figure 17:**
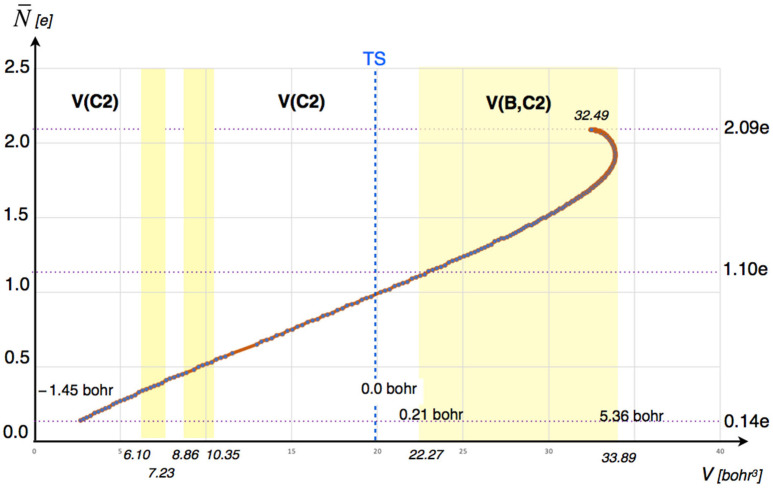
The change in electron population associated with the formation of the B-C2 bond, observed through the V(C2) and V(B) or V(B,C2) basins (yellow area), as a function of their volume (V). In TS the V(B,C2) basin is observed. The first point on the graph corresponds to a population of 0.14e for the V(C2) basin at the beginning of phase III. Analysis was performed using DFT(M062X) data.

The nature of the B-C bond requires additional clarification. In phase IV, two attractors and non-bonding basins, V(C2) and V(B), are observed. However, in later phases and in the reaction product, a bonding attractor and basin, V(B,C2), appear. This raises the question: from which point on the IRC path, after the annihilation of the V(B) attractor, does the topological ELF analysis show the presence of the V(B,C2) attractor and basin instead of V(C2)? In the graph illustrating the relationship between the population of the V(C2) or V(B,C2) basin and its volume, V (see Figure 17), the points on the IRC path where either the V(C2) or V(B,C2) attractors are observed are marked. The V(B,C2) attractor and basin are continuously observed after the reacting system passes through the TS for points on the IRC path with a reaction coordinate ranging from 0.21 bohr to 5.36 bohr (the last point on the IRC path). Unfortunately, in the case of donor–acceptor bonds, such as B-N, there are numerical challenges in properly describing the synapticity of the basin [42]. Depending on the distance between the N and B atoms, the topological analysis of ELF may show either the monosynaptic attractor and basin V(N) or the disynaptic attractor and basin V(B,N). This interpretation is relevant for the results obtained for two ranges of reaction coordinates (from −1.08 to −0.96 bohr and from −0.80 to −0.73 bohr) in phases III (6 points) and IV (5 points), where instead of the V(C2) attractor, the ELF analysis indicates the presence of the V(B,C2) attractor.

Despite these interpretational difficulties, the V(B,C2) attractor and disynaptic basin are first observed in phase VII, which is interpreted as the formation of the B-C2 bond. In Figure 12, the modified Lewis formula represents the boron-carbon bond in phase VII with a continuous line (B-C2). The “saturation” of the V(B,C2) basin with electron density continues until the last point on the IRC path, as illustrated by the graph in Figure 17. Interestingly, it is observed that in the final part of phase X, the volume of the V(B,C2) basin decreases while its electron population steadily increases.

**Figure 18:**
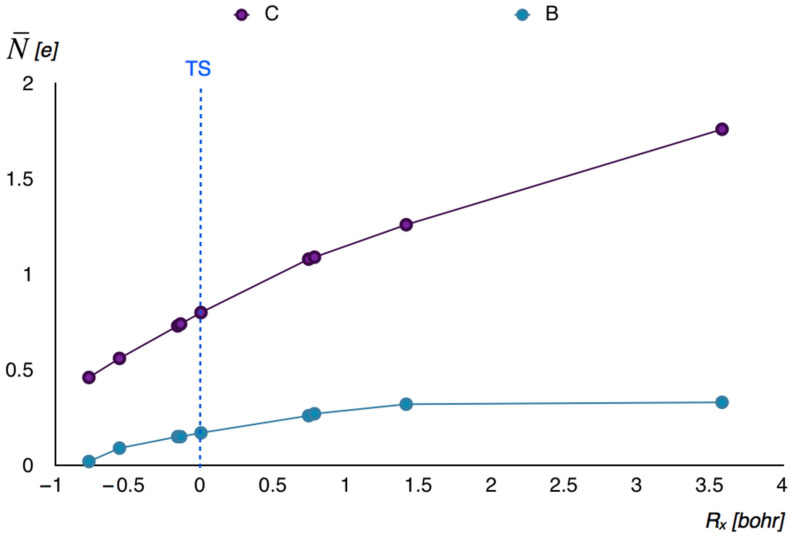
Contribution of the populations of quantum atoms B and C2 to the populations of localization basins V(C2) and V(B) or V(B,C2) involved in the formation of the B-C2 bond, as a function of the reaction coordinate, R_x_. Lines connecting the points are included to highlight the trends of changes. Analysis was performed using DFT(M062X) data.

The second aspect of the nature of the B-C bond that requires comment concerns the contributions of the quantum atoms B and C, specifically the polarity of the bond. In the initial phase of B-C2 bond formation, an attractor and basin V(C2) are formed, characterized exclusively by the valence electron density of the C atom. This raises the question: what is the mechanism behind the sharing of electron density between the B and C atoms? It is known that in both py→BC_5_H_5_ and py→BBa, the contribution of the quantum atom B is 16–17%. To address this, the contribution of the B quantum atom to the populations of the V(C2) and V(B,C2) basins was investigated for selected points along the IRC path, with the results presented in Figure 18. The calculations show that, although the contribution of the B quantum atom is small relative to that of the C quantum atom, it begins from the very start of the B-C2 bond formation and continues through both the V(C2) and V(B,C2) basins. Therefore, the idea that electron density from the C atom is initially concentrated in the C2⋯B region, with the B atom contributing only at a later stage, is not supported. The covalent nature of the B-C bond is instead associated with the sharing of electron density by both atoms from the outset, beginning in phase III.

The distances r(C1,C7) and r(C2,B), at which the C1-C7 bond is formed, are 2.047 Å and 1.985 Å, respectively. For the B-C2 bond, depending on whether the beginning of its formation is considered to be the moment of annihilation of the V(B) basin (phase V) or the point at which only the disynaptic V(B,C2) basin is observed (phase VII), the distances are 2.344 Å, 2.258 Å, or 2.232 Å, 2.149 Å, respectively.

**Figure 19:**
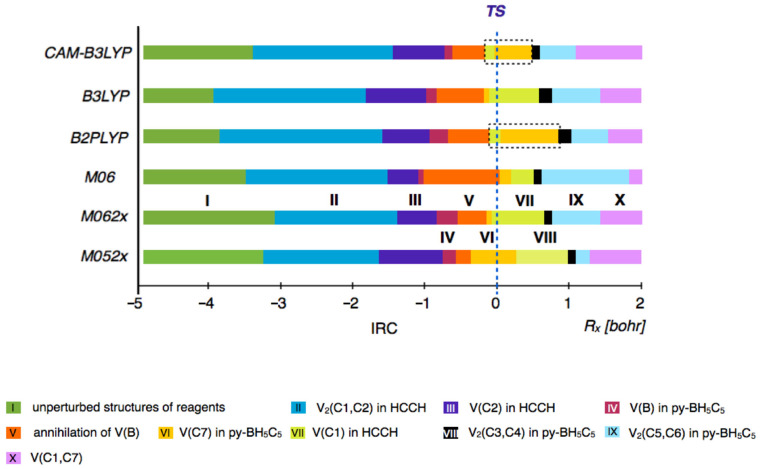
Barcodes illustrating the length of individual reaction phases (number of points on the IRC path) depending on the applied electron density functional. Horizontal axis—reaction coordinate R_x_. Phases whose order is changed with respect to DFT(M062X) results are marked with a dashed line.

Since the reaction mechanism of py→BC_5_H_5_ and HC≡CH was investigated using the DFT method with six electron density functionals (M052X, M062X, M06, B2PLYP, B3LYP, and CAM-B3LYP), there is an opportunity to describe their influence on the BET results. The reference will be the results obtained using the DFT(M062X) method, as described above. A comparison of the barcodes illustrating the length of the individual phases (number of points on the IRC path) depending on the applied electron density functional is shown in Figure 19. Due to the different lengths of the initial (I) and final (X) phases, their barcodes were arbitrarily adjusted to R_x_ values of −4.97 and 2.0 bohr, respectively.

The most interesting question, of course, is whether a change in the choice of electron density functional affects the order of the phases and, consequently, the description of the reaction mechanism, as well as the length of the phases (a number of points). The answer to the first question is affirmative; the choice of functional can indeed have a qualitative impact on the description of the reaction mechanism. An example can be seen in the results obtained for the CAM-B3LYP and B2PLYP functionals, which “show” that the order of formation of the intermolecular C1-C7 bond differs from that in the case of results obtained using the B3LYP functional and functionals from the Truhlar group (see Figure 19). The DFT(M062X) results analyzed above indicate that in phase VI, the V(C7) attractor is formed, and in phase VII, the V(C1) attractor. Therefore, bond formation begins in the py→BC_5_H_5_ molecule. However, calculations using CAM-B3LYP and B2PLYP functionals show the opposite order: first, the V(C1) basin is formed in HC≡CH, followed by the V(C7) basin in py→BC_5_H_5_. While this might not seem to be a significant change from the perspective of chemical bond evolution, it remains an observed fact, suggesting that in other studies, for different systems and using different computational methods, the obtained results (reaction mechanisms) may differ qualitatively and quantitatively.

The answer to the second question is also affirmative. The lengths of the individual reaction phases are different and can also differ qualitatively. For example, phase V in calculations using the M06 functional is clearly longer than phases VI and VII, which differs from the results obtained by the DFT(M052X), where phase V is shorter than both V and VII. There are also similarities; all methods clearly show that phase II, in which the reorganization of electron density at the C1≡C2 bond occurs, is the longest. Similarly, phase VIII, which is associated with the formation of the second V_2_(C3,C4) basin in the BC_5_ ring, is very short in most cases. Taking the transition state structure as a distinguished point on the IRC path, it can be seen that it is associated with different SSDs depending on the functional used. In the reference DFT(M062X) calculations, the TS is observed in phase VII, and similarly in B3LYP and CAM-B3LYP calculations (though the order of phases is changed). Interestingly, in DFT(M06) calculations, the TS is observed relatively “early” in phase V. TS in phase VI is shown by DFT(M052X) and B2PLYP calculations. There is no doubt that the use of different functionals changes the characteristics of the reaction mechanism quantitatively and, in special cases, also qualitatively.

## 4. Materials and Methods

All calculations have been performed using the DFT method with the B3LYP [44], CAM-B3LYP [49], M052X [46], M06 [45], M062X [45], and B2PLYP [47] electron density functionals, as coded in the GAUSSIAN09 (G16, rev C.01) program [52]. The electrons have been described using the 6-311++G(d,p) basis set from G16 [53,54,55]. Minima on the potential energy surface have been confirmed by the absence of imaginary vibrational frequencies. Zero-point vibrational energy (ΔZPVE) corrections were included in the energy calculations, i.e., reaction energy (ΔE_r_), and activation energy (ΔE_a_). The interaction energy (E_int_), calculated as the difference between the MC energy and the energy of isolated monomers with geometry from the MC, has been corrected for BSSE (E_int_^CP^).

The reaction path has been calculated using the intrinsic reaction coordinate (IRC) [56] standard procedure (the HPC algorithm) [57] in both directions with a step size of 0.01 bohr. The net reaction coordinate (R_x_) values before reaching the transition state structure are presented in the text using negative values. Initial force constants were calculated at the beginning of the IRC calculation (CalcFC).

Lewis structure drawings were prepared using MarvinSketch, version 16.12.5.0, provided by ChemAxon Ltd. (Budapest, Hungary) [58].

Topological analysis of ELF was performed using the TopMod09 program [59] over a rectangular parallelepiped grid with a step size of 0.05 bohr. The wave function (closed-shell monodeterminantal SCF) for the topological analysis was calculated in single-point calculations for optimized geometric structures. No symmetry was applied. In the case of the B2PLYP calculations, natural orbitals and their occupations for the current density were used to generate the wfn files. For the M052X results, the stability of the wave function was tested for all points on the IRC path, and no instabilities were detected.

All ELF field catastrophes described in the paper were identified by analyzing the distances between critical points (CPs) involved in the ELF topology change, as well as by examining the evolution of the Hessian matrix determinant, |det(H)|, calculated for the analyzed CPs at points on the IRC path before and after the catastrophe. The procedure is detailed in the paper [37]. For the analyzed reaction, the precise description of fold catastrophe identification is provided in one case—specifically, for the last two phases, IX and X. In this case, the change in the ELF molecular field topology is interpreted as the formation of the C1-C7 bond through the annihilation of the attractor V(C7) and the critical point (3,−1), followed by the change in synapticity from the V(C1) basin to the V(C1,C7) basin.

The notation F^+^ and F, in the case of fold catastrophes, refers to an increase (F^+^) or decrease (F) in the number of attractors.

Visualization of the ELF function was performed using the VMD program for macOS X86, version 1.9.3 (30 November 2016) [60] and the MultiWFN program [61]. The localization basins for the transition state structure were obtained using MultiWFN for electron densities greater than 0.001 au. It is worth noting that the ELF in the region of the C≡C bond in the acetylene fragment is characterized by three V_i=1..3_(C,C) attractors and basins, while only one such attractor and basin is localized using the TopMod09 program.

## 5. Conclusions

Analysis of the DA reaction mechanism between py→BC_5_H_5_ and HC≡CH, performed using a combined topological analysis of the molecular ELF field and elements of catastrophe theory, reveals a picture consistent with general chemical understanding. The B-C and C-C chemical bonds are formed as a result of electron density transfer from neighboring bonds to regions where new bonds are expected. This process is reflected by ELF field catastrophes and the formation of local attractors and ELF basins.

It should be emphasized that the B-C2 and C1-C7 bonds are not formed simultaneously along the IRC path. Qualitative changes in the B-C bond occur between phases III and VII, whereas the “construction” of the C1-C7 bond takes place from phases VI to X.

In the case of the C-C bond, its formation mechanism closely resembles that previously described in the study of the reaction between 1,3-butadiene and ethylene [35]. Initially, two non-bonding attractors and basins, V(C1) and V(C7), are observed. Subsequently, the V(C7) attractor is annihilated, and V(C1) is “transformed” into a bonding basin, V(C1,C7). In contrast, differences arise in the case of the B-C bond, likely due to its specific nature. As shown by calculations for the isolated molecules py→BC_5_H_5_ and py→BBa, only 16–17% of the electron density in the B-C bond originates from the B atom. In the early stages of bond formation (phases III and IV), two non-bonding basins, V(C2) and V(B), are observed, which supports the classification of the B-C bond as a covalent–polarized bond. However, the V(B) basin has a negligible population (0.01–0.03e) and is annihilated relatively quickly (phase IV, after 8 points on the IRC path). This extremely low population of the V(B) basin raises an important question: to what extent can the B-C bond be considered a covalent-polarized bond, if it reflects only minimal shared electron density from the valence shells of the B and C atoms?

If the moment of B-C bond formation is defined as the point at which the attractor and basin V(C2) change their synaptic type from monosynaptic (non-bonding) to disynaptic (bonding), forming V(B,C2), then the B-C bond is established after the reacting system passes the transition state structure, at r(C1,C7) and r(C2,B) distances of 2.232 and 2.149 Å, respectively. On the other hand, a single attractor and a single basin V(C2) are observed at distances of 2.344 and 2.258 Å (prior to the TS), and this point on the IRC path may also be considered as the initial stage of B-C bond formation.

The analysis of the influence of the functional on the BET results showed that its construction can indeed impact the description of the reaction mechanism. For example, in the case of the CAM-B3LYP and B2PLYP functionals, the order of formation of the nonbonding monosynaptic basins V(C1) and V(C7)—which mark the onset of the intermolecular C1-C7 bond—is reversed compared to results obtained using B3LYP, M06, M062X, and M052X. Nevertheless, the comparison reveals that the overall mechanism, from the perspective of chemical bond evolution, is largely consistent regardless of the functional applied. Although the lengths of individual phases may differ, the same sequence of events is observed: first, changes in electron localization occur within the C≡C bond of the HC≡CH molecule (phases I and II); next, the first B-C bond forms (phases III–V or III–VII); this is followed by the formation of the second C-C bond (phases VI–X); and finally, the C^…^C bonds within the BC_5_ ring are transformed into localized C=C double bonds (phases VIII and IX). Of course, it should be emphasized that these steps represent continuous changes in the electron density throughout the reaction pathway.

I believe that the studies described above helped clarify the mechanism of the DA reaction, particularly by highlighting key differences in the nature of the C-C and B-C bonds.

## Figures and Tables

**Table 1:** Optimized bond lengths, r(C1,C7) and r(C2,B) (in Å), between py→BC_5_H_5_ and HC≡CH molecules in the reaction transition state structure, calculated using different electron density functionals.

Bond Length/Funct.	B3LYP	CAM-B3LYP	M052X	M06	M062X	B2PLYP
r(C1,C7)	2.290	2.270	2.247	2.352	2.266	2.307
r(C2,B)	2.153	2.186	2.221	2.076	2.175	2.132

**Table 2:** Populations (in e) of the core and valence localization basins in the HC≡CH molecule, calculated using different DFT functionals.

Basin/Funct.	B3LYP	CAM-B3LYP	M052X	M06	M062X	B2PLYP
C(C)	2.10	2.09	2.09	2.10	2.09	2.09
V(H,C)	2.34	2.34	2.34	2.33	2.32	2.35
V(C,C)	5.11	5.13	5.11	5.14	5.17	5.11

**Table 3:** Electron populations (in e) for selected localization basins in phases I to X of the py→BC_5_H_5_ + HC≡CH→py→BBa reaction. The values correspond to the initial points on the IRC path for each phase, with IRC coordinates R_x_ given in bohr. For comparison, data for the isolated reactants, the product, and the transition state structure are also included.

Param./Phase:	Reactants	I	II	III	IV	V	VI	VII	VII TS	VIII	IX	X	Product
R_x_		−6.38	−3.12	−1.45	−0.77	−0.56	−0.16	−0.14	0	0.74	0.78	1.40	
V(N)	2.55	2.58	2.57	2.55	2.54	2.53	2.52	2.52	2.52	2.50	2.50	2.48	2.44
V(C1,C7)	-	-	-	-	-	-	-	-	-	-	-	1.15 ^(d)^	1.93
V(C1)	-	-	-	-	-	-	-	0.23	0.26	0.49	0.50	-	-
V(C7)	-	-	-	-	-	-	0.12	0.12	0.17	0.36	0.38	-	-
V(B,C2)	-	-	-	-	-	-	-	-	0.98	1.36	1.37	1.59	2.15
V(B)	-	-	-	-	0.01	-	-	-	-	-	-	-	-
V(C2)	-	-	-	0.14	0.48	0.65	0.89	0.91	-	-	-	-	-
V_1_(C1,C2)	5.17	5.25 ^(a)^ {	1.80	2.48	2.41	2.35	2.25	2.24	2.21	2.05	2.04	1.94	1.70
V_2_(C1,C2)			3.42	2.57	2.36	2.31	2.22	2.22	2.19	2.04	2.04	1.94	1.70
V_1_(C3,C4)	2.70	2.72 ^(b)^	2.77	2.88	2.95	2.97	3.02	3.02	3.03 {	1.50	1.49	1.54	1.70
V_2_(C3,C4)										1.64	1.66	1.67	1.70
V_1_(C5,C6)	2.69	2.74 ^(c)^	2.78	2.88	2.95	2.97	3.02	3.03	3.04	3.13 {	1.52	1.55	1.70
V_2_(C5,C6)											1.63	1.66	1.70

^(a)^ single attractor and basin V_1_(C1,C2) in HC≡CH (reactant), ^(b)^ single attractor and basin V_1_(C3,C4) in py→BC_5_H_5_, ^(c)^ single attractor and basin V_1_(C5,C6) in py→BC_5_H_5_ (reactant), and ^(d)^ single attractor and basin V(C1,C7) in py→BBa (product), the atom numbers are shown in Figure 4 for py→BC_5_H_5_ and Figure 7 for py→BBa.

## Data Availability

Data is contained within the article or Supplementary Material.

## Supplementary Materials

The following supporting information can be downloaded at: https://www.mdpi.com/article/10.3390/molecules30112357/s1, Figure S1: (a) The core and valence attractors of ELF molecular field in the cyclohexene molecule, (b) Lewis formula for the cyclohexene molecule.

## Abbreviations

The following abbreviations are used in this manuscript.

BET	Bonding evolution theory
SSD	Structure stability domain
DFT	Density functional theory
ELF	Electron localization function
CP	Critical point
DA	Diels–Alder
G16	Gaussian16
IRC	Intrinsic reaction coordinate
CT	Catastrophe theory
PT2	Second-order perturbation theory
F	Fold catastrophe
MC	Molecular complex
