# Supplementary figures and images for: The Mechanism of Boron–Carbon Bond Formation in the DA Reaction of the Pyridine Adduct of Borabenzene with Acetylene: A Topological Analysis of the ELF Function and Catastrophe Theory
Source: Molecules. 2025 May 28;30(11):2357. doi: 10.3390/molecules30112357 (PMC12156250; doi:10.3390/molecules30112357)

a)

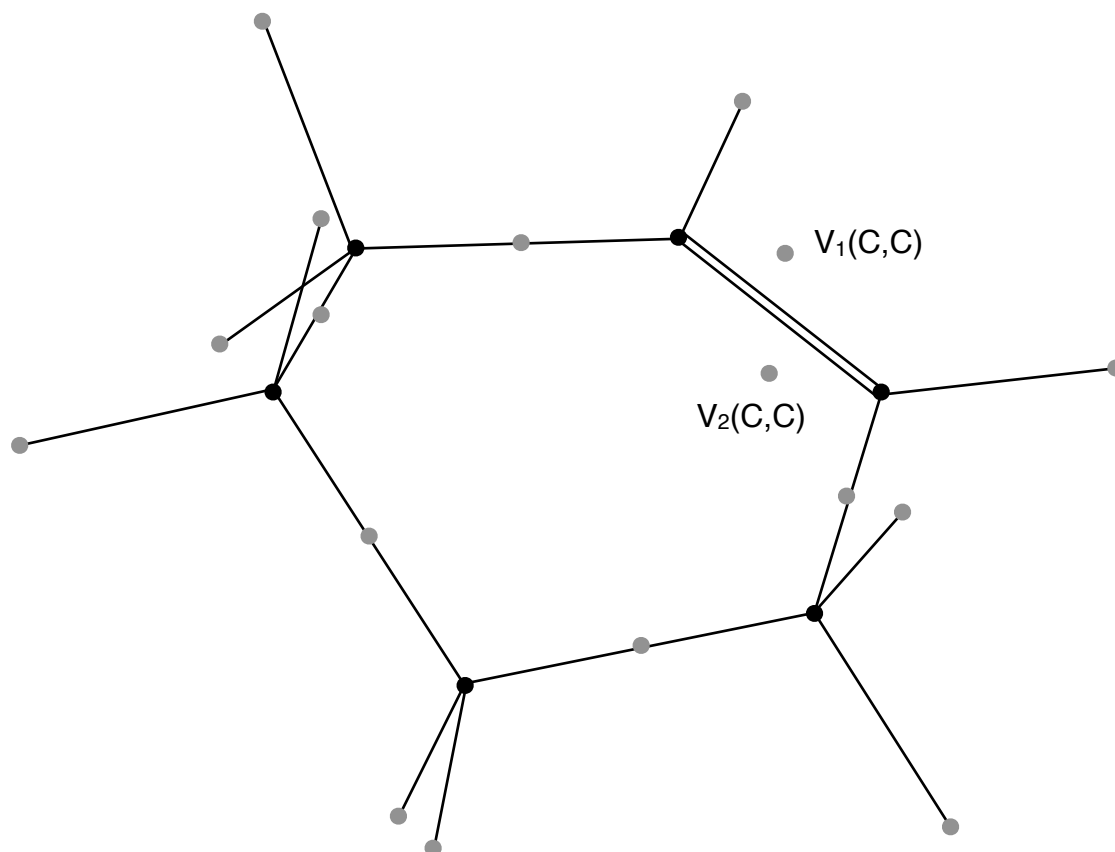

b)

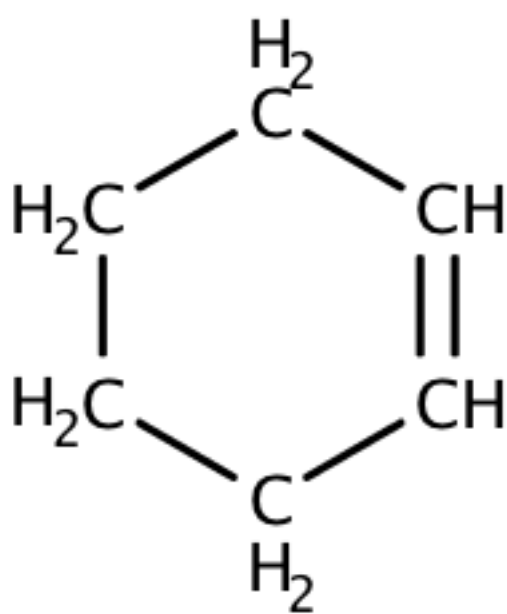

Supplement: Supplementary file 1 [file molecules-30-02357-s001.zip › molecules-3563583-supplementary.pdf]
